# Supplementary material for: Immune transcriptomic differences in paediatric patients with SARS-CoV-2 compared to other lower respiratory tract infections
Source: bioRxiv. 2025 Nov 7:2025.11.07.687132. Preprint. [Version 1] doi: 10.1101/2025.11.07.687132 (PMC12637703; doi:10.1101/2025.11.07.687132)
Supplement: Supplement 10 [file media-10.docx]

# Supplementary data

# Supplementary data

Supplementary Fig 1 Module dendrogram

Supplementary Fig 2 Distribution of genes per module

Supplementary Fig 3 Venn diagram shared modules between respiratory infections

Supplementary Fig 4 REVIGO biological process for correlated modules with LRTI

Supplementary Fig 5 Blood composition comparisons between LRTI

Supplementary Fig 6 Shared cell type

Supplementary Fig 7 Shared severity predictors for LRTI

Supplementary table S1 Respiratory infection TWAS FDR< 0.05

Supplementary table S2 Respiratory infection TWAS enrichment

Supplementary table S3 WGCNA module genes and eigengene

Supplementary table S4 Network degree distribution for module correlated to LRTI

Supplementary table S5 Enrichment for module correlated to LRTI

Supplementary Table S6 Cell type proportions

Supplementary table S7 severity predictors

Supplementary table S8 Drug target look-up

Supplementary table S9 Severity predictors and target prioritization

Supplementary file Modules_Network_analysis.cys.
