# Supplementary material for: Immune transcriptomic differences in paediatric patients with SARS-CoV-2 compared to other lower respiratory tract infections
Source: bioRxiv. 2025 Nov 7:2025.11.07.687132. Preprint. [Version 1] doi: 10.1101/2025.11.07.687132 (PMC12637703; doi:10.1101/2025.11.07.687132)
Supplement: 1 [file NIHPP2025.11.07.687132V1-supplement-1.pdf]

bioRxiv preprint doi: https://doi.org/10.1101/2025.11.07.687132; this version posted November 7, 2025. The copyright holder for this preprint (which was not certified by peer review) is the author/funder, who has granted bioRxiv a license to display the preprint in perpetuity. It is made available under aCC-BY 4.0 International license.

Supplementary data

Supplementary Fig 1 Module dendrogram

Supplementary Fig 2 Distribution of genes per module

Supplementary Fig 3 Venn diagram shared modules between respiratory infections

Supplementary Fig 4 REVIGO biological process for correlated modules with LRTI

Supplementary Fig 5 Blood composition comparisons between LRTI

Supplementary Fig 6 Shared cell types

Supplementary Fig 7 Shared severity predictors for LRTI

Supplementary table S1 Respiratory infection TWAS FDR< 0.05

Supplementary table S2 Respiratory infection TWAS enrichment

Supplementary table S3 WGCNA module genes and eigengene
Supplementary table S4 Network degree distribution for module correlated to LRTI

Supplementary table S5 Enrichment for module correlated to LRTI

Supplementary Table S6 Cell type proportions

Supplementary table S7 severity predictors

Supplementary table S8 Drug target look-up

Supplementary table S9 Severity predictors and target prioritization

Supplementary file Modules_Network_analysis.cys.

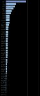

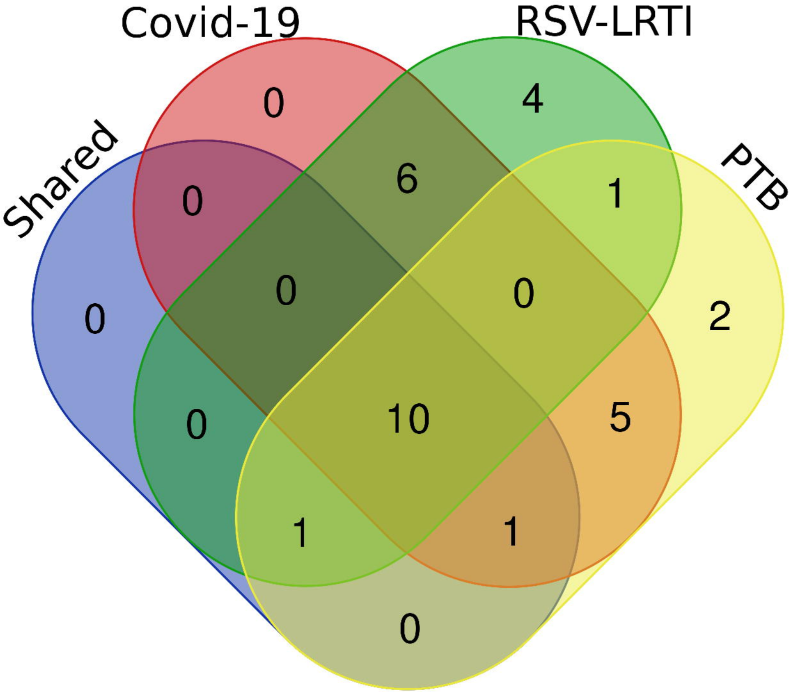
Covid-19
RSV-LRTI
Shared
PTB
0
4
6
0
1
0
0
0
2
0
10
5
1
1
0

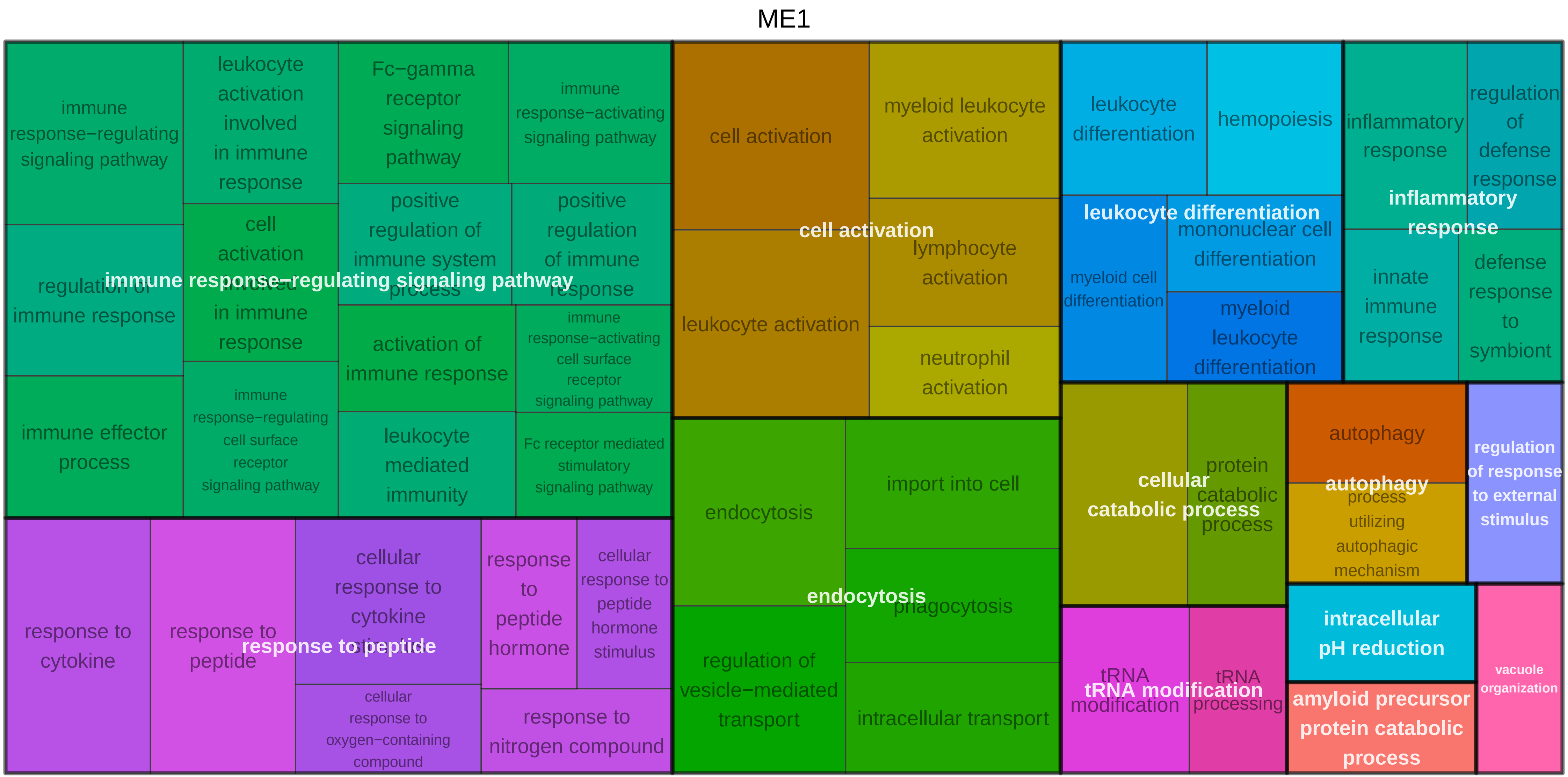
ME1
immune response−regulating signaling pathway
immune response−regulating signaling pathway
leukocyte activation involved in immune response
Fc−gamma receptor signaling pathway
immune response−activating signaling pathway
regulation of immune response
cell activation involved in immune response
positive regulation of immune system process
positive regulation of immune response
activation of immune response
immune response−activating cell surface receptor signaling pathway
immune effector process
immune response−regulating cell surface receptor signaling pathway
leukocyte mediated immunity
Fc receptor mediated stimulatory signaling pathway
cell activation
cell activation
myeloid leukocyte activation
lymphocyte activation
leukocyte activation
neutrophil activation
leukocyte differentiation
leukocyte differentiation
hemopoiesis
mononuclear cell differentiation
myeloid cell differentiation
myeloid leukocyte differentiation
inflammatory response
inflammatory response
regulation of defense response
innate immune response
defense response to symbiont
response to cytokine
response to peptide
response to peptide
cellular response to cytokine stimulus
response to peptide hormone
cellular response to peptide hormone stimulus
cellular response to oxygen−containing compound
response to nitrogen compound
endocytosis
endocytosis
import into cell
phagocytosis
regulation of vesicle−mediated transport
intracellular transport
cellular catabolic process
protein catabolic process
autophagy
autophagy
process utilizing autophagic mechanism
regulation of response to external stimulus
tRNA modification
tRNA modification
tRNA processing
intracellular pH reduction
amyloid precursor protein catabolic process
vacuole organization

ME10

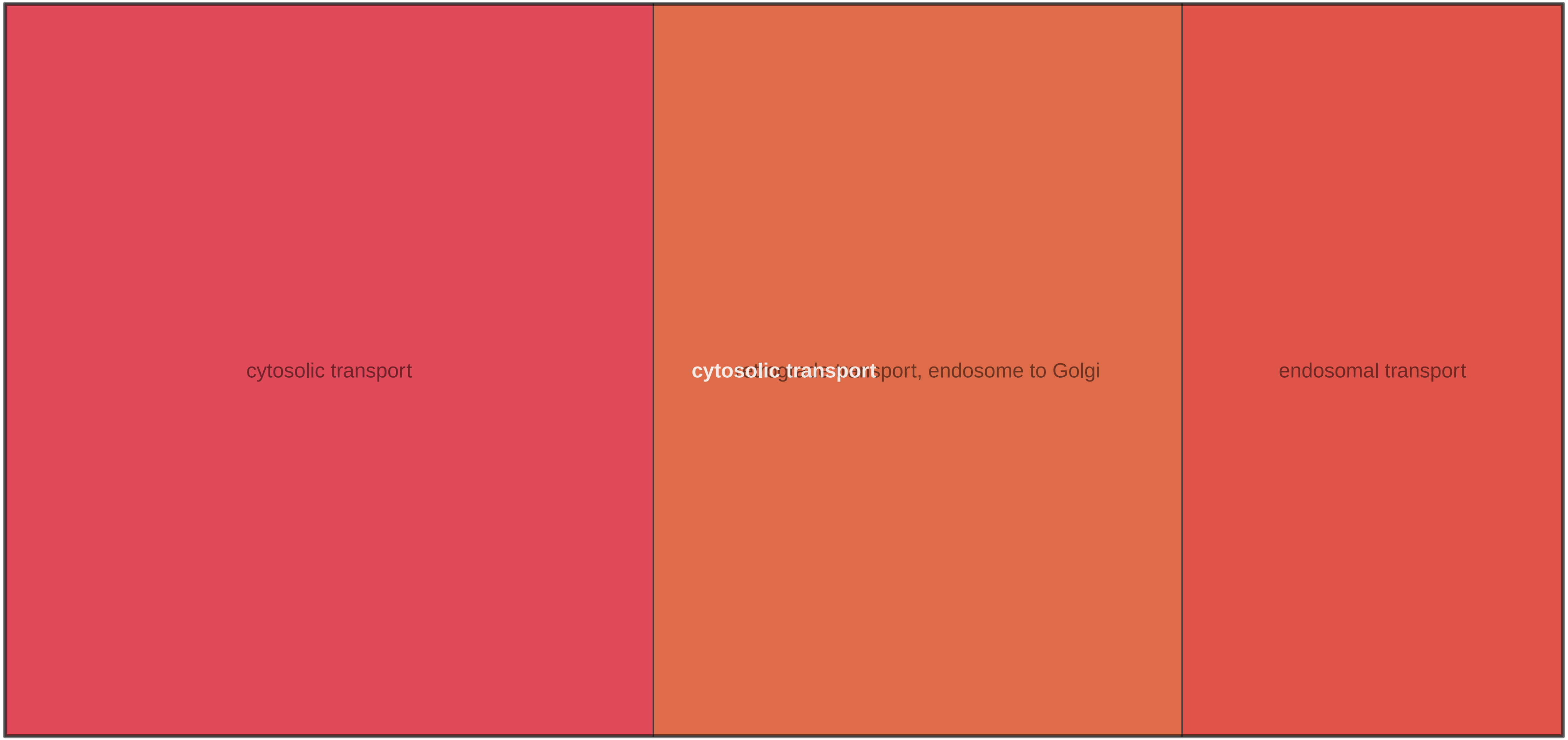
cytosolic transport
cytosolic transport
retrograde transport, endosome to Golgi
endosomal transport

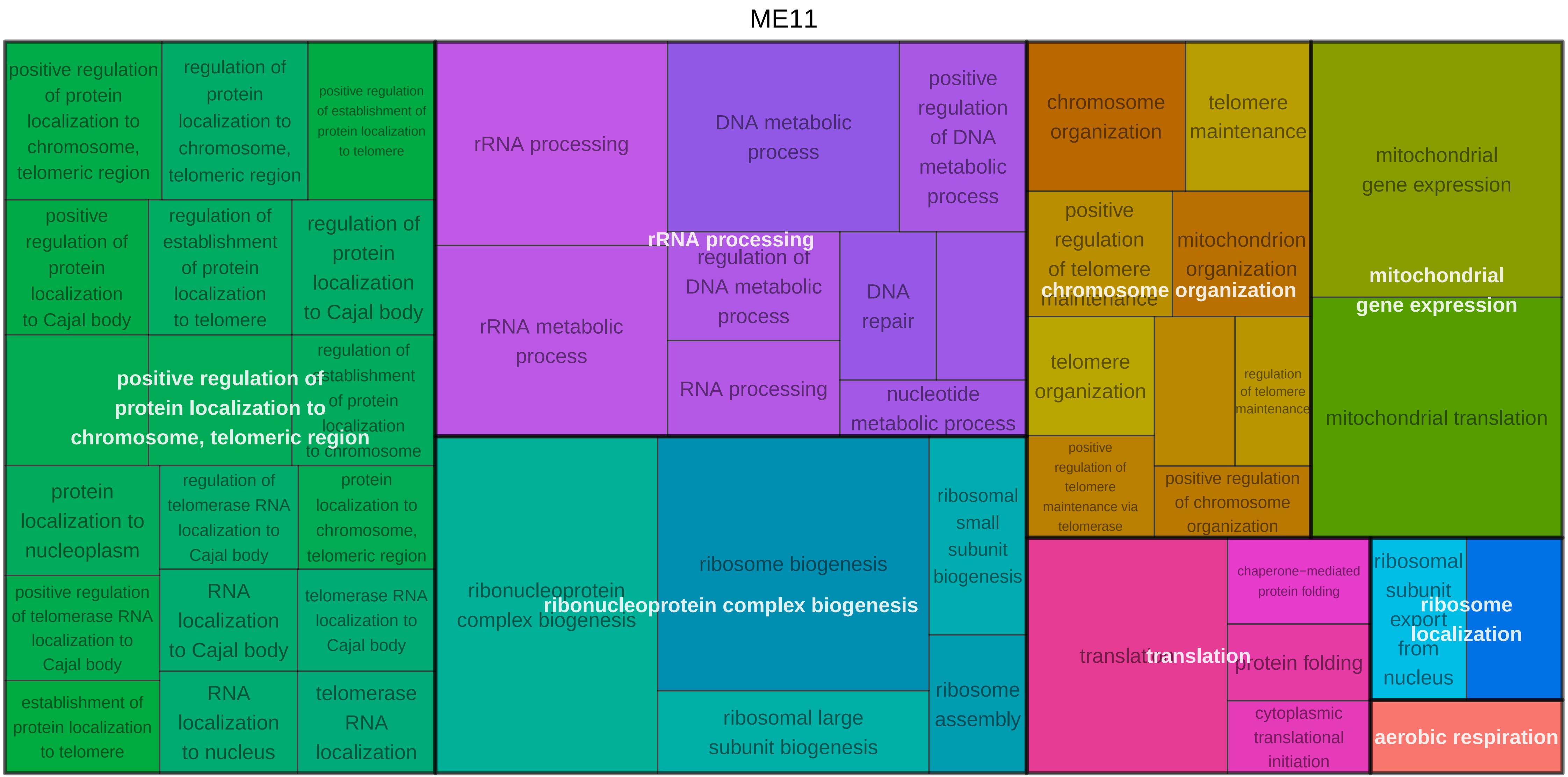
ME11
positive regulation of protein localization to chromosome, telomeric region
regulation of protein localization to chromosome, telomeric region
positive regulation of establishment of protein localization to telomere
positive regulation of protein localization to Cajal body
regulation of establishment of protein localization to telomere
regulation of protein localization to Cajal body
positive regulation of protein localization to chromosome, telomeric region
regulation of establishment of protein localization to chromosome
protein localization to nucleoplasm
regulation of telomerase RNA localization to Cajal body
protein localization to chromosome, telomeric region
positive regulation of telomerase RNA localization to Cajal body
RNA localization to Cajal body
telomerase RNA localization to Cajal body
establishment of protein localization to telomere
RNA localization to nucleus
telomerase RNA localization
rRNA processing
DNA metabolic process
positive regulation of DNA metabolic process
rRNA processing
regulation of DNA metabolic process
DNA repair
rRNA metabolic process
RNA processing
nucleotide metabolic process
ribonucleoprotein complex biogenesis
ribosome biogenesis
ribosomal small subunit biogenesis
ribonucleoprotein complex biogenesis
ribosome assembly
ribosomal large subunit biogenesis
chromosome organization
telomere maintenance
positive regulation of telomere maintenance
mitochondrion organization
chromosome organization
telomere organization
regulation of telomere maintenance
positive regulation of telomere maintenance via telomerase
positive regulation of chromosome organization
mitochondrial gene expression
mitochondrial gene expression
mitochondrial translation
translation
chaperone−mediated protein folding
translation
protein folding
cytoplasmic translational initiation
ribosomal subunit export from nucleus
ribosome localization
aerobic respiration

ME12

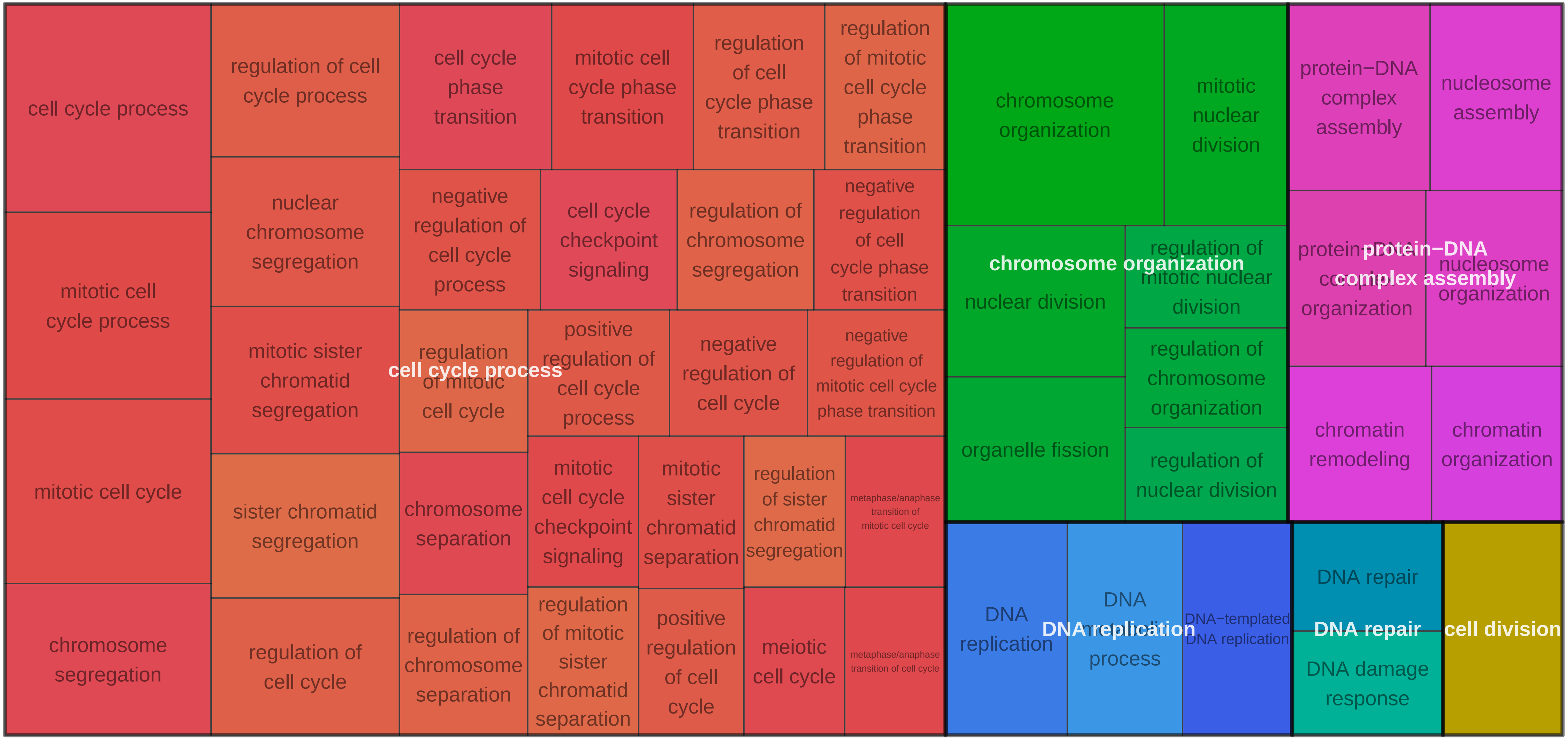
cell cycle process
regulation of cell cycle process
cell cycle phase transition
mitotic cell cycle phase transition
regulation of cell cycle phase transition
regulation of mitotic cell cycle phase transition
nuclear chromosome segregation
negative regulation of cell cycle process
cell cycle checkpoint signaling
regulation of chromosome segregation
negative regulation of cell cycle phase transition
mitotic cell cycle process
mitotic sister chromatid segregation
regulation of mitotic cell cycle
cell cycle process
positive regulation of cell cycle process
negative regulation of cell cycle
negative regulation of mitotic cell cycle phase transition
mitotic cell cycle
sister chromatid segregation
chromosome separation
mitotic cell cycle checkpoint signaling
mitotic sister chromatid separation
regulation of sister chromatid segregation
metaphase/anaphase transition of mitotic cell cycle
chromosome segregation
regulation of cell cycle
regulation of chromosome separation
regulation of mitotic sister chromatid separation
positive regulation of cell cycle
meiotic cell cycle
metaphase/anaphase transition of cell cycle
chromosome organization
mitotic nuclear division
chromosome organization
nuclear division
regulation of mitotic nuclear division
organelle fission
regulation of chromosome organization
regulation of nuclear division
protein−DNA complex assembly
nucleosome assembly
protein−DNA complex assembly
nucleosome organization
chromatin remodeling
chromatin organization
DNA replication
DNA replication
DNA metabolic process
DNA−templated DNA replication
DNA repair
DNA repair
DNA damage response
cell division

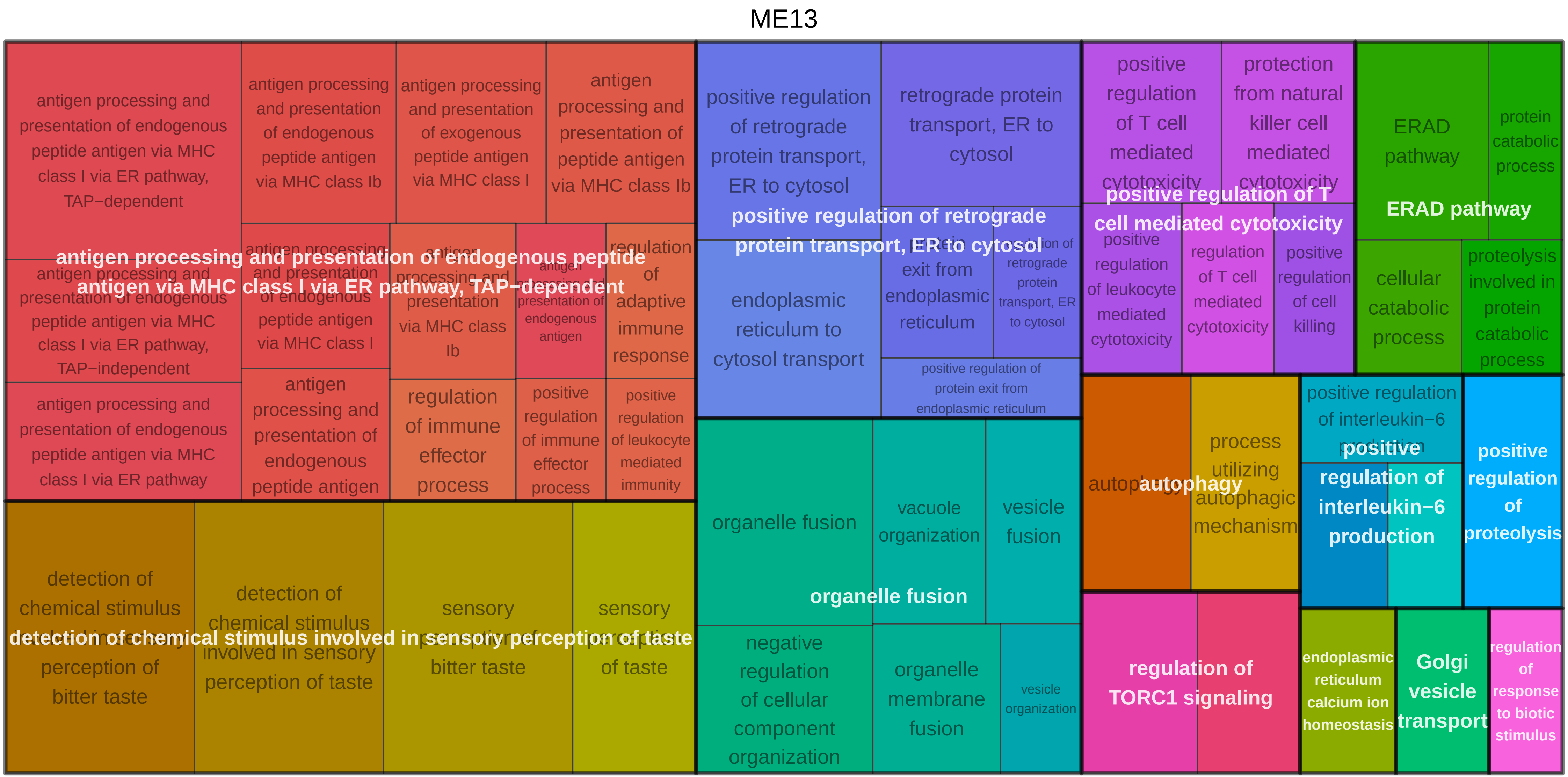
ME13
antigen processing and presentation of endogenous peptide antigen via MHC class I via ER pathway, TAP−dependent
antigen processing and presentation of endogenous peptide antigen via MHC class Ib
antigen processing and presentation of exogenous peptide antigen via MHC class I
antigen processing and presentation of peptide antigen via MHC class Ib
antigen processing and presentation of endogenous peptide antigen via MHC class I via ER pathway, TAP−independent
antigen processing and presentation of endogenous peptide antigen via MHC class I
presentation via MHC class Ib
presentation of endogenous antigen
regulation of adaptive immune response
antigen processing and presentation of endogenous peptide antigen via MHC class I via ER pathway
antigen processing and presentation of endogenous peptide antigen
regulation of immune effector process
positive regulation of immune effector process
positive regulation of leukocyte mediated immunity
antigen processing and presentation of endogenous peptide antigen via MHC class I via ER pathway, TAP−dependent
positive regulation of retrograde protein transport, ER to cytosol
retrograde protein transport, ER to cytosol
positive regulation of retrograde protein transport, ER to cytosol
endoplasmic reticulum to cytosol transport
exit from endoplasmic reticulum
retrograde protein transport, ER to cytosol
positive regulation of protein exit from endoplasmic reticulum
positive regulation of T cell mediated cytotoxicity
protection from natural killer cell mediated cytotoxicity
positive regulation of T cell mediated cytotoxicity
positive regulation of leukocyte mediated cytotoxicity
regulation of T cell mediated cytotoxicity
positive regulation of cell killing
ERAD pathway
protein catabolic process
ERAD pathway
cellular catabolic process
proteolysis involved in protein catabolic process
detection of chemical stimulus
perception of bitter taste
detection of chemical stimulus involved in sensory perception of taste
sensory
bitter taste
sensory
of taste
detection of chemical stimulus involved in sensory perception of taste
organelle fusion
vacuole organization
vesicle fusion
organelle fusion
negative regulation of cellular component organization
organelle membrane fusion
vesicle organization
autophagy
process utilizing autophagic mechanism
positive regulation of interleukin−6 production
positive regulation of interleukin−6 production
positive regulation of proteolysis
regulation of TORC1 signaling
endoplasmic reticulum calcium ion homeostasis
Golgi vesicle transport
regulation of response to biotic stimulus

ME14

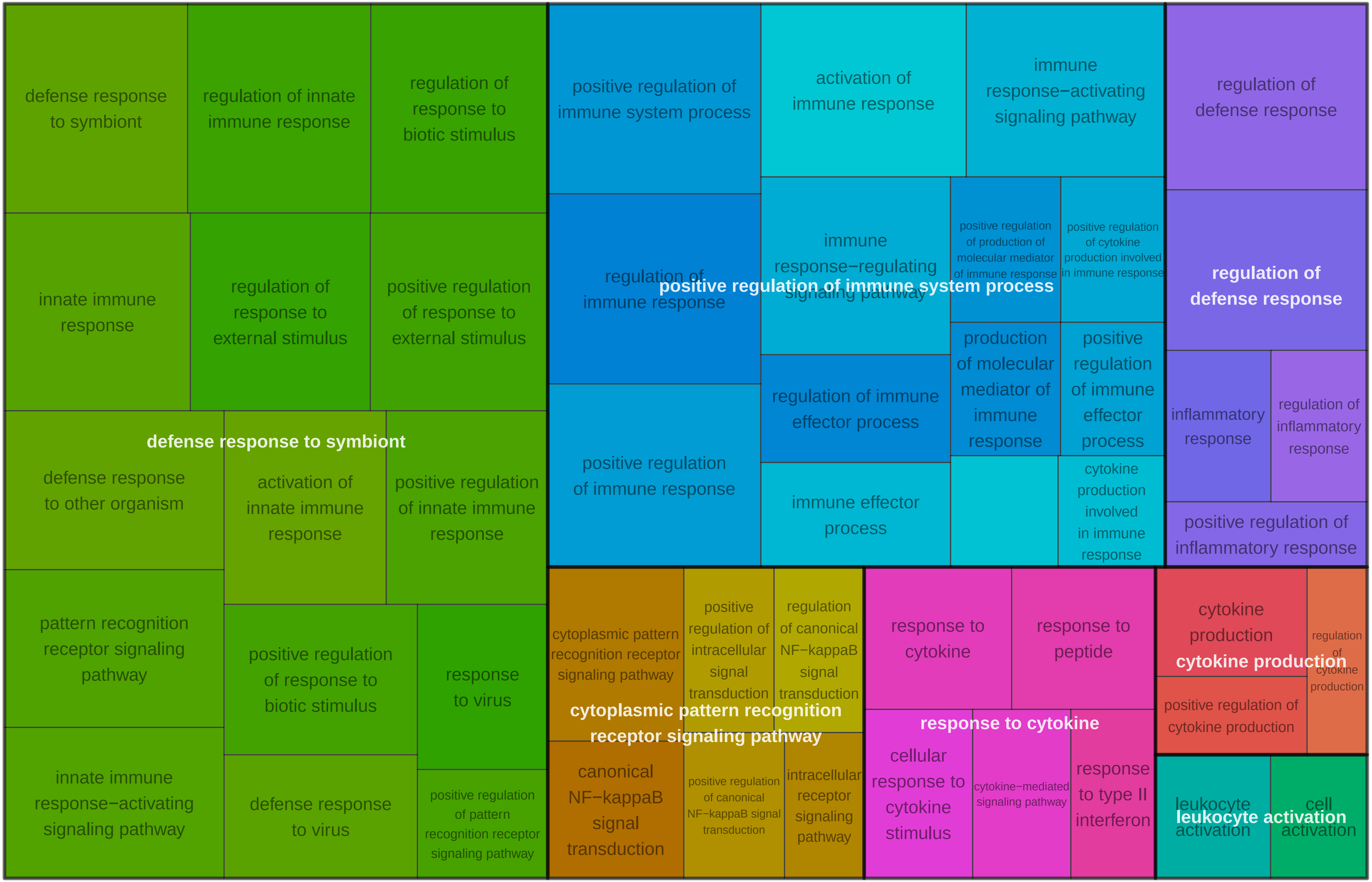
defense response to symbiont
regulation of innate immune response
regulation of response to biotic stimulus
innate immune response
regulation of response to external stimulus
positive regulation of response to external stimulus
defense response to symbiont
defense response to other organism
activation of innate immune response
positive regulation of innate immune response
pattern recognition receptor signaling pathway
positive regulation of response to biotic stimulus
response to virus
innate immune response−activating signaling pathway
defense response to virus
positive regulation of pattern recognition receptor signaling pathway
positive regulation of immune system process
activation of immune response
immune response−activating signaling pathway
regulation of immune response
immune response−regulating signaling pathway
positive regulation of production of molecular mediator of immune response
positive regulation of cytokine production involved in immune response
positive regulation of immune system process
regulation of immune effector process
production of molecular mediator of immune response
positive regulation of immune effector process
positive regulation of immune response
immune effector process
cytokine production involved in immune response
regulation of defense response
regulation of defense response
inflammatory response
regulation of inflammatory response
positive regulation of inflammatory response
cytoplasmic pattern recognition receptor signaling pathway
positive regulation of intracellular signal transduction
regulation of canonical NF−kappaB signal transduction
cytoplasmic pattern recognition receptor signaling pathway
canonical NF−kappaB signal transduction
positive regulation of canonical NF−kappaB signal transduction
intracellular receptor signaling pathway
response to cytokine
response to peptide
response to cytokine
cellular response to cytokine stimulus
cytokine−mediated signaling pathway
response to type II interferon
cytokine production
cytokine production
regulation of cytokine production
positive regulation of cytokine production
leukocyte activation
leukocyte activation
cell activation

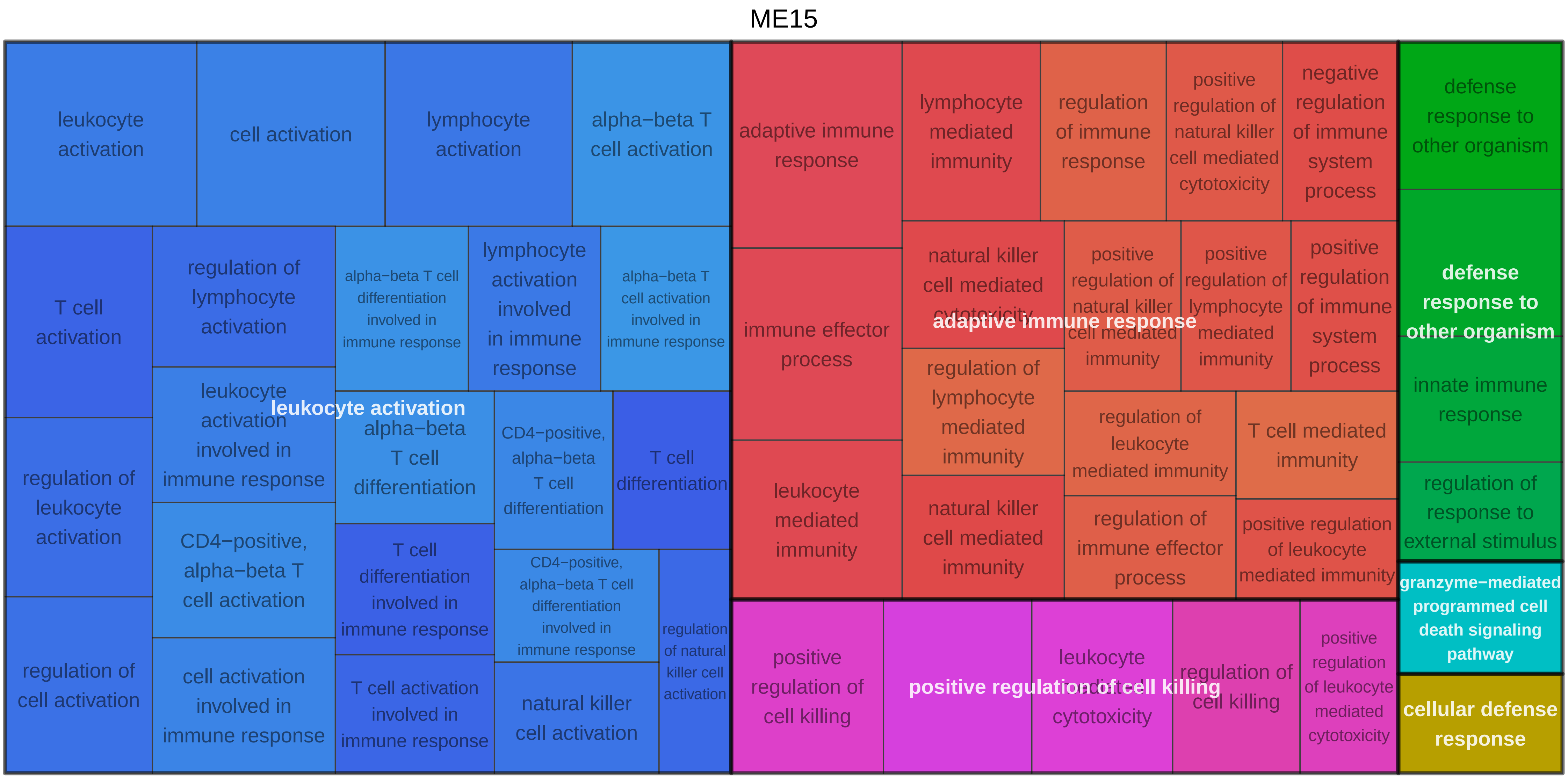
ME15
leukocyte activation
cell activation
lymphocyte activation
alpha−beta T cell activation
T cell activation
regulation of lymphocyte activation
alpha−beta T cell differentiation involved in immune response
lymphocyte activation involved in immune response
alpha−beta T cell activation involved in immune response
leukocyte activation involved in immune response
leukocyte activation
alpha−beta T cell differentiation
CD4−positive, alpha−beta T cell differentiation
T cell differentiation
regulation of leukocyte activation
CD4−positive, alpha−beta T cell activation
T cell differentiation involved in immune response
CD4−positive, alpha−beta T cell differentiation involved in immune response
regulation of natural killer cell activation
regulation of cell activation
cell activation involved in immune response
T cell activation involved in immune response
natural killer cell activation
adaptive immune response
lymphocyte mediated immunity
regulation of immune response
positive regulation of natural killer cell mediated cytotoxicity
negative regulation of immune system process
natural killer cell mediated cytotoxicity
positive regulation of natural killer cell mediated immunity
positive regulation of lymphocyte mediated immunity
positive regulation of immune system process
adaptive immune response
immune effector process
regulation of lymphocyte mediated immunity
regulation of leukocyte mediated immunity
T cell mediated immunity
leukocyte mediated immunity
natural killer cell mediated immunity
regulation of immune effector process
positive regulation of leukocyte mediated immunity
positive regulation of cell killing
positive regulation of cell killing
leukocyte mediated cytotoxicity
regulation of cell killing
positive regulation of leukocyte mediated cytotoxicity
defense response to other organism
defense response to other organism
innate immune response
regulation of response to external stimulus
granzyme−mediated programmed cell death signaling pathway
cellular defense response

ME16

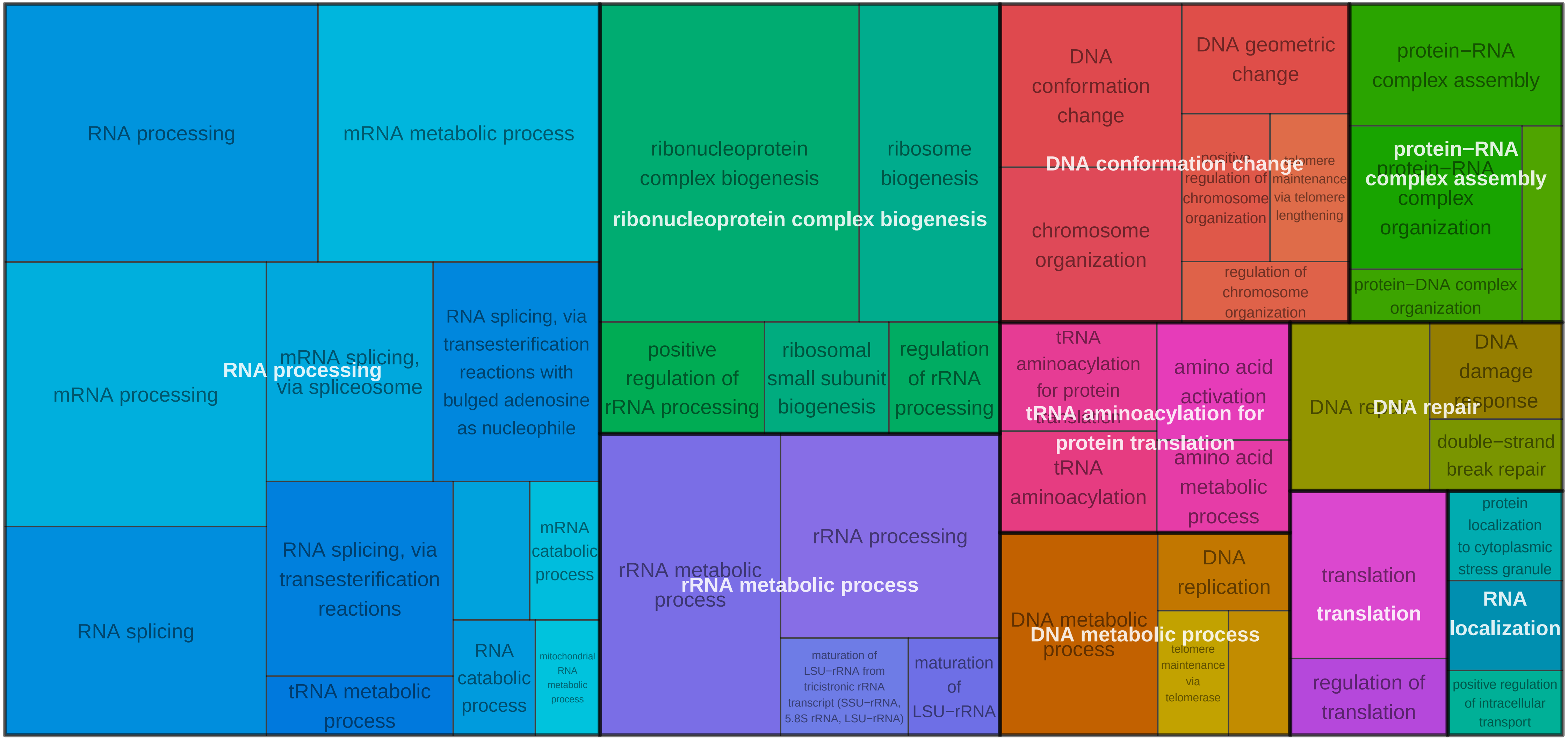
RNA processing
mRNA metabolic process
mRNA processing
mRNA splicing, via spliceosome
RNA splicing, via transesterification reactions with bulged adenosine as nucleophile
RNA splicing
RNA splicing, via transesterification reactions
tRNA metabolic process
mRNA catabolic process
RNA catabolic process
mitochondrial RNA metabolic process
ribonucleoprotein complex biogenesis
ribosome biogenesis
positive regulation of rRNA processing
ribosomal small subunit biogenesis
regulation of rRNA processing
rRNA metabolic process
rRNA processing
maturation of LSU−rRNA from tricistronic rRNA transcript (SSU−rRNA, 5.8S rRNA, LSU−rRNA)
maturation of LSU−rRNA
DNA conformation change
DNA geometric change
chromosome organization
positive regulation of chromosome organization
telomere maintenance via telomere lengthening
regulation of chromosome organization
protein−RNA complex assembly
protein−RNA complex organization
protein−DNA complex organization
tRNA aminoacylation for protein translation
amino acid activation
tRNA aminoacylation
amino acid metabolic process
DNA repair
DNA damage response
double−strand break repair
DNA metabolic process
DNA replication
telomere maintenance via telomerase
translation
regulation of translation
protein localization to cytoplasmic stress granule
RNA localization
positive regulation of intracellular transport

ME18

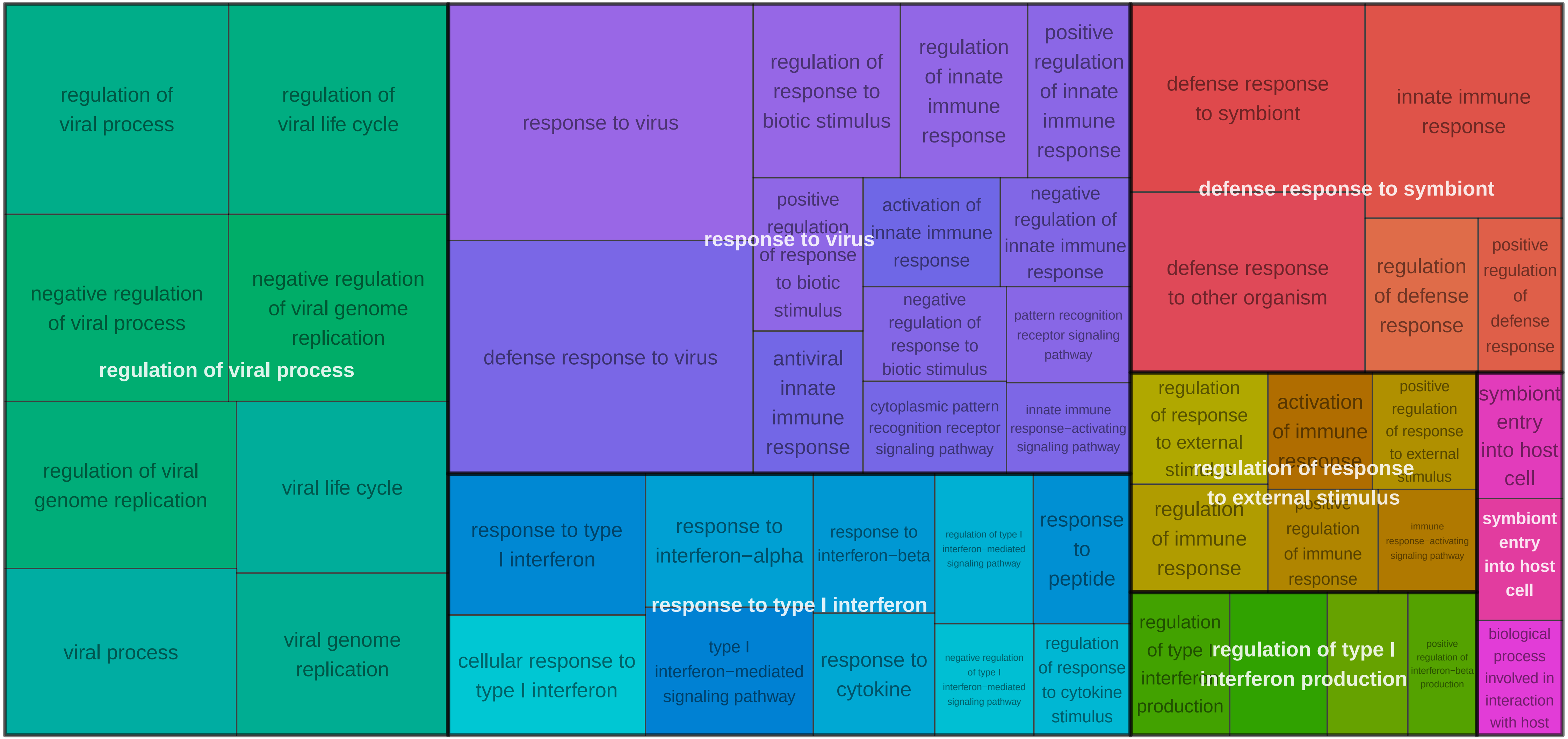

regulation of viral process
regulation of viral life cycle
negative regulation of viral process
negative regulation of viral genome replication
regulation of viral process
regulation of viral genome replication
viral life cycle
viral process
viral genome replication
response to virus
regulation of response to biotic stimulus
regulation of innate immune response
positive regulation of innate immune response
positive regulation of response to biotic stimulus
response to virus
activation of innate immune response
negative regulation of innate immune response
defense response to virus
negative regulation of response to biotic stimulus
pattern recognition receptor signaling pathway
antiviral innate immune response
cytoplasmic pattern recognition receptor signaling pathway
innate immune response−activating signaling pathway
defense response to symbiont
innate immune response
defense response to symbiont
defense response to other organism
regulation of defense response
positive regulation of defense response
regulation of response to external stimulus
activation of immune response
positive regulation of response to external stimulus
regulation of response to external stimulus
symbiont entry into host cell
regulation of immune response
positive regulation of immune response
immune response−activating signaling pathway
symbiont entry into host cell
response to type I interferon
response to interferon−alpha
response to interferon−beta
regulation of type I interferon−mediated signaling pathway
response to peptide
response to type I interferon
cellular response to type I interferon
type I interferon−mediated signaling pathway
response to cytokine
negative regulation of type I interferon−mediated signaling pathway
regulation of response to cytokine stimulus
regulation of type I interferon production
regulation of type I interferon production
positive regulation of interferon−beta production
biological process involved in interaction with host

ME2

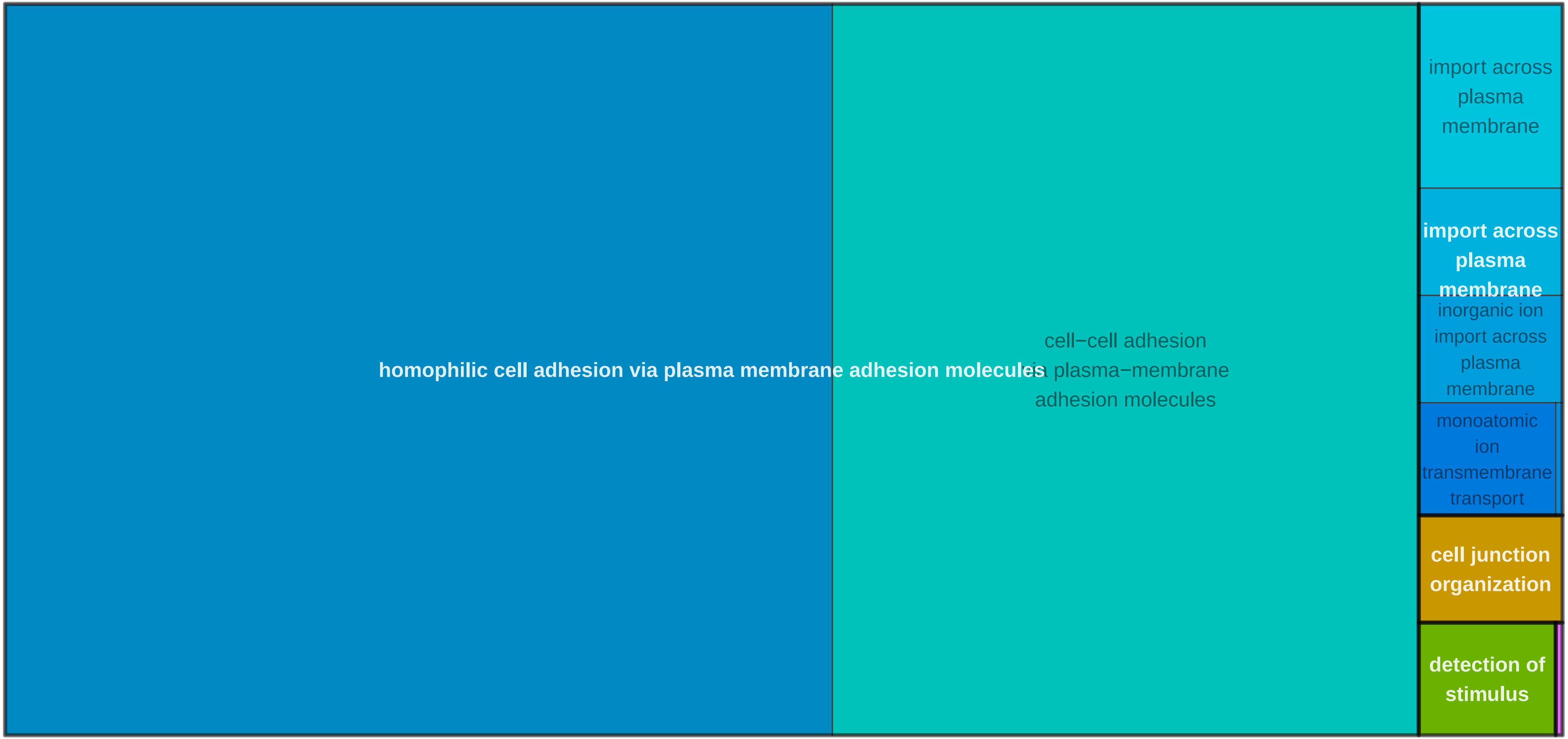
homophilic cell adhesion via plasma membrane adhesion molecules
cell−cell adhesion via plasma−membrane adhesion molecules
import across plasma membrane
import across plasma membrane
inorganic ion import across plasma membrane
monoatomic ion transmembrane transport
cell junction organization
detection of stimulus

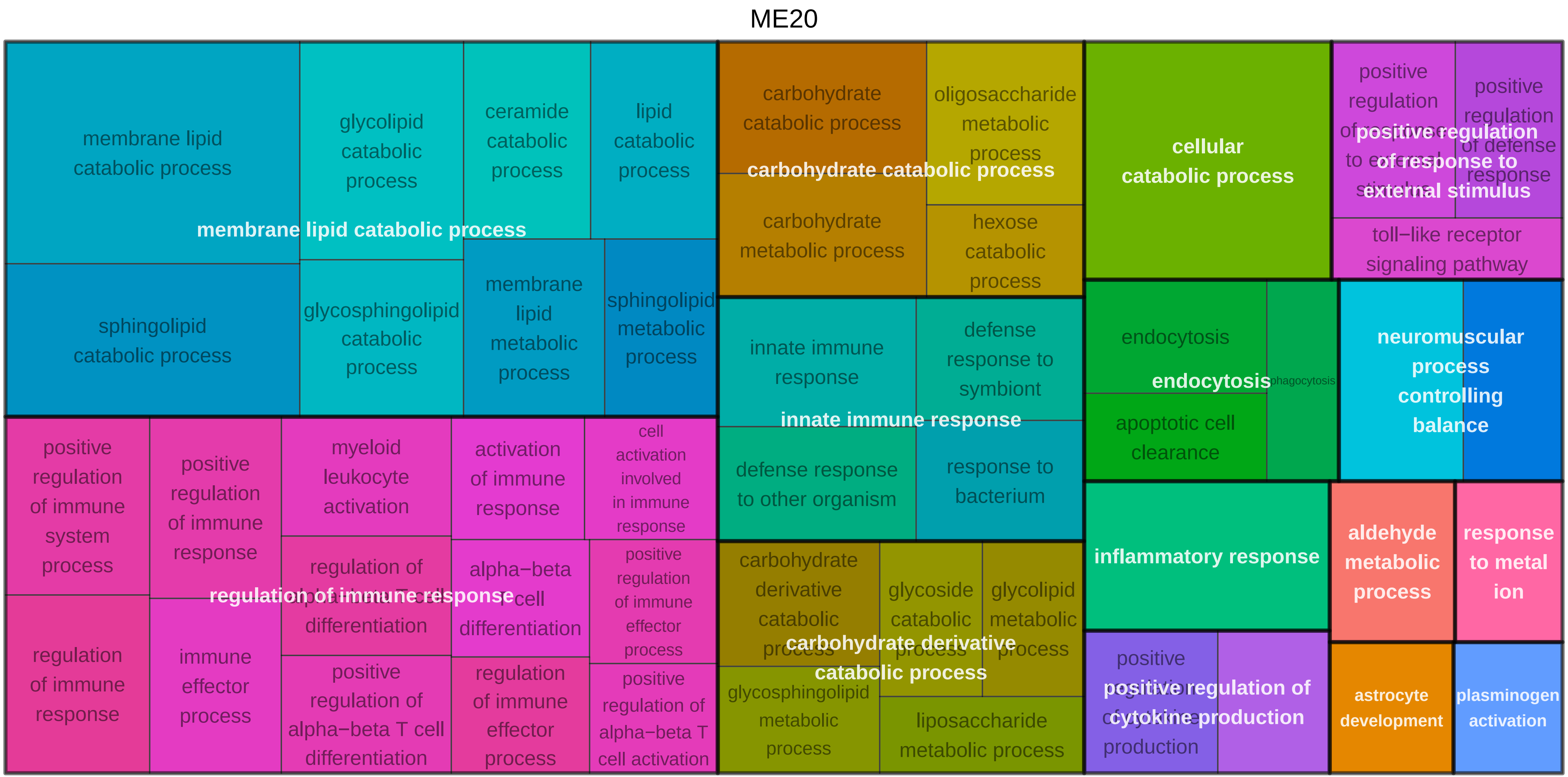

ME20
membrane lipid catabolic process
membrane lipid catabolic process
glycolipid catabolic process
ceramide catabolic process
lipid catabolic process
sphingolipid catabolic process
glycosphingolipid catabolic process
membrane lipid metabolic process
sphingolipid metabolic process
carbohydrate catabolic process
oligosaccharide metabolic process
carbohydrate catabolic process
carbohydrate metabolic process
hexose catabolic process
cellular catabolic process
positive regulation of response to external stimulus
positive regulation of defense response
toll−like receptor signaling pathway
innate immune response
defense response to symbiont
innate immune response
defense response to other organism
response to bacterium
endocytosis
endocytosis
phagocytosis
apoptotic cell clearance
neuromuscular process controlling balance
positive regulation of immune system process
positive regulation of immune response
myeloid leukocyte activation
activation of immune response
cell activation involved in immune response
regulation of immune response
alpha−beta T cell differentiation
positive regulation of immune effector process
regulation of immune response
immune effector process
positive regulation of alpha−beta T cell differentiation
regulation of immune effector process
positive regulation of alpha−beta T cell activation
carbohydrate derivative catabolic process
glycoside catabolic process
glycolipid metabolic process
carbohydrate derivative catabolic process
glycosphingolipid metabolic process
liposaccharide metabolic process
inflammatory response
aldehyde metabolic process
response to metal ion
positive regulation of cytokine production
astrocyte development
plasminogen activation

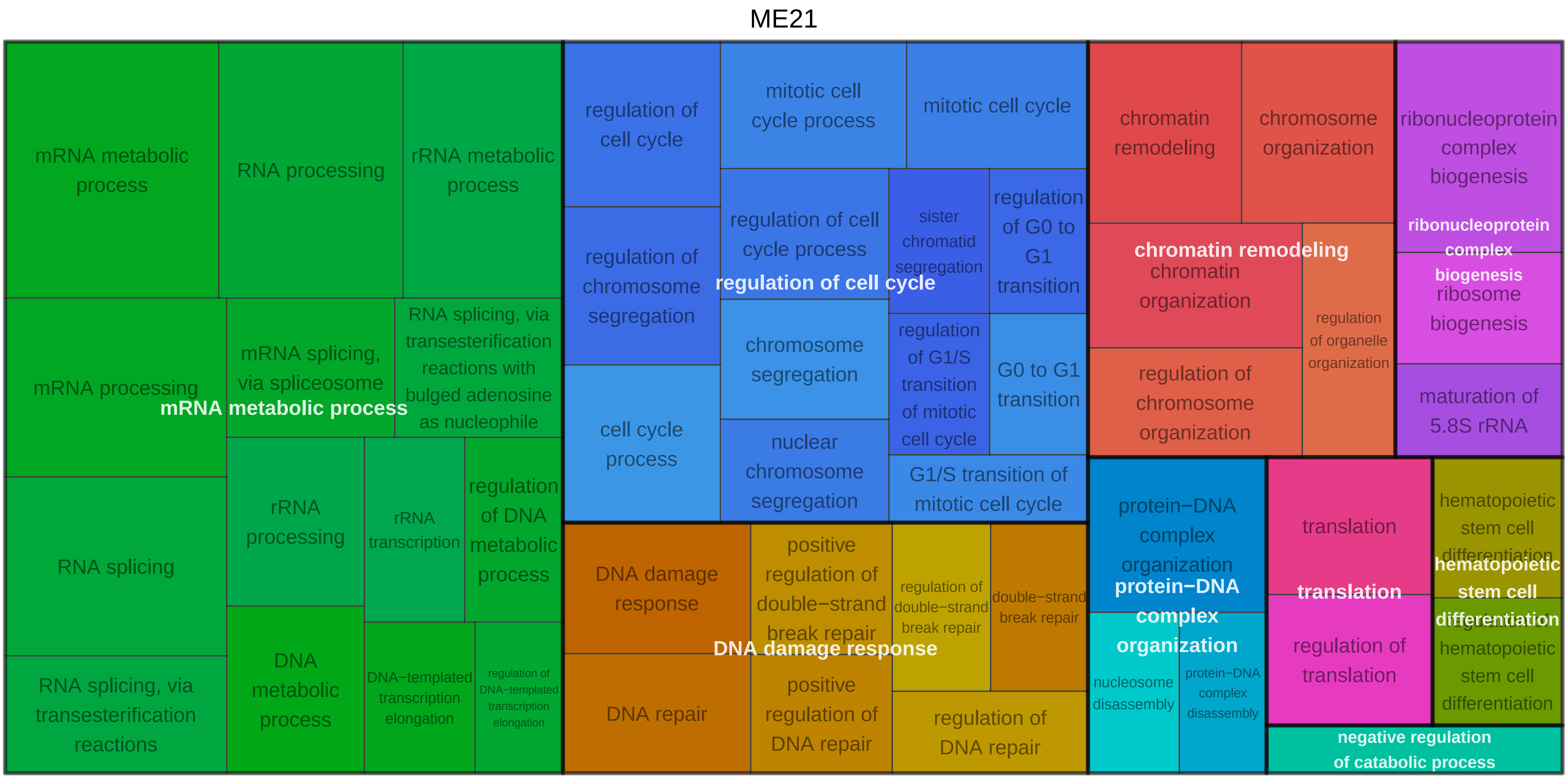
ME21
mRNA metabolic process
RNA processing
rRNA metabolic process
mRNA processing
mRNA splicing, via spliceosome
RNA splicing, via transesterification reactions with bulged adenosine as nucleophile
mRNA metabolic process
RNA splicing
rRNA processing
rRNA transcription
regulation of DNA metabolic process
RNA splicing, via transesterification reactions
DNA metabolic process
DNA−templated transcription elongation
regulation of DNA−templated transcription elongation
regulation of cell cycle
mitotic cell cycle process
mitotic cell cycle
regulation of cell cycle process
sister chromatid segregation
regulation of G0 to G1 transition
regulation of chromosome segregation
regulation of cell cycle
chromosome segregation
regulation of G1/S transition of mitotic cell cycle
G0 to G1 transition
cell cycle process
nuclear chromosome segregation
G1/S transition of mitotic cell cycle
DNA damage response
positive regulation of double−strand break repair
regulation of double−strand break repair
double−strand break repair
DNA damage response
DNA repair
positive regulation of DNA repair
regulation of DNA repair
chromatin remodeling
chromosome organization
chromatin remodeling
chromatin organization
regulation of organelle organization
regulation of chromosome organization
ribonucleoprotein complex biogenesis
ribonucleoprotein complex biogenesis
ribosome biogenesis
maturation of 5.8S rRNA
protein−DNA complex organization
protein−DNA complex organization
nucleosome disassembly
protein−DNA complex disassembly
translation
translation
regulation of translation
hematopoietic stem cell differentiation
hematopoietic stem cell differentiation
regulation of hematopoietic stem cell differentiation
negative regulation of catabolic process

ME22

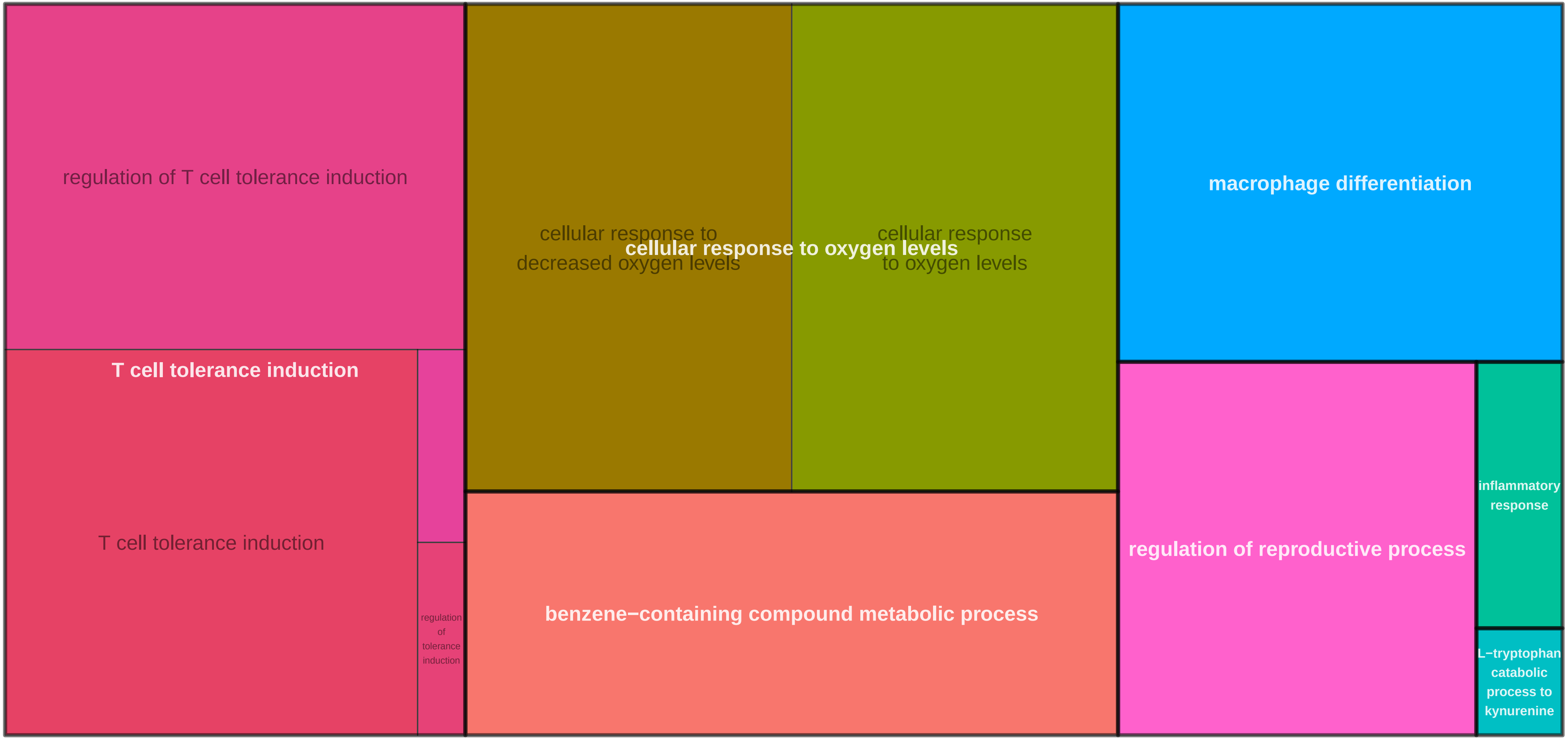
regulation of T cell tolerance induction
T cell tolerance induction
T cell tolerance induction
regulation of tolerance induction
cellular response to decreased oxygen levels
cellular response to oxygen levels
cellular response to oxygen levels
benzene−containing compound metabolic process
macrophage differentiation
regulation of reproductive process
inflammatory response
L−tryptophan catabolic process to kynurenine

ME24

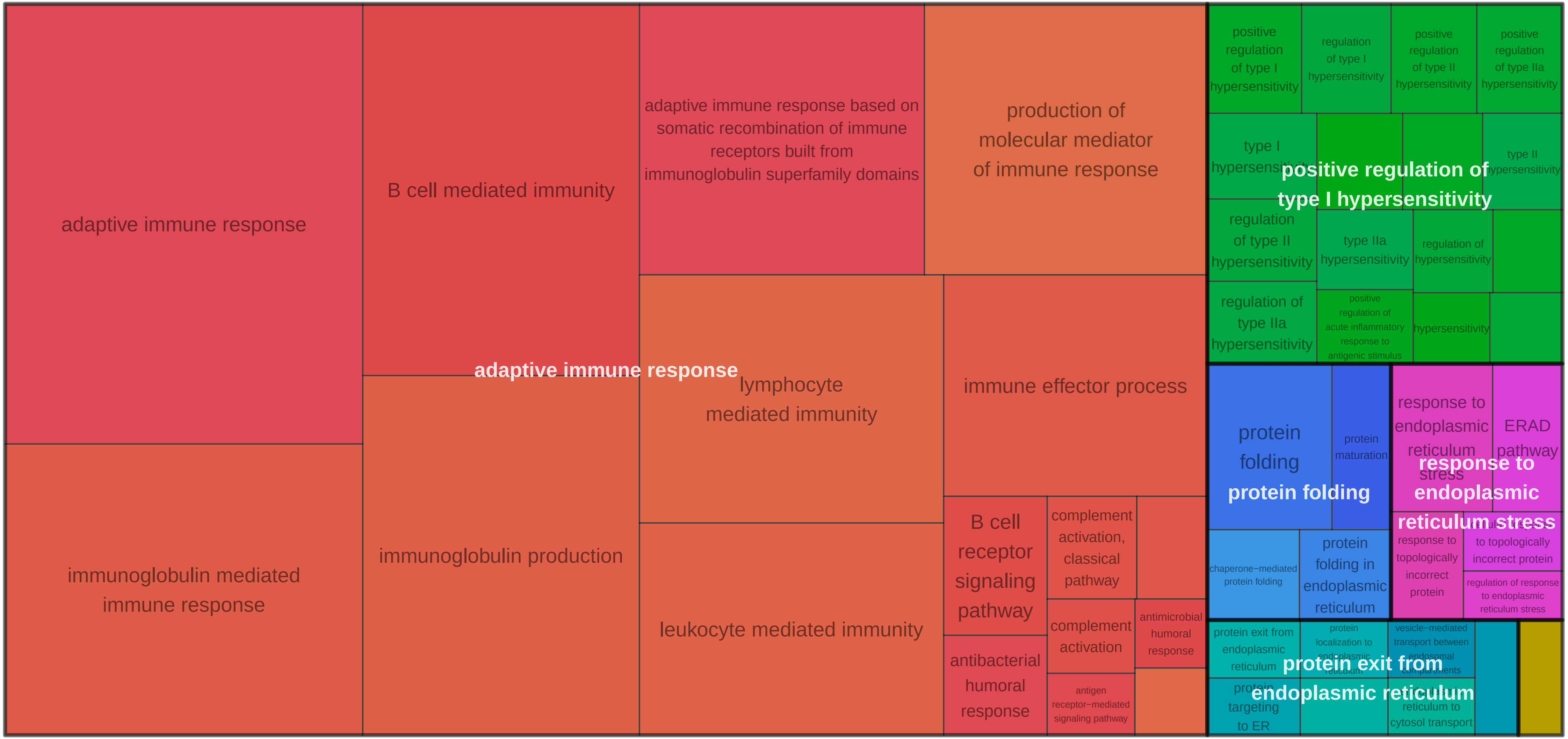

adaptive immune response
B cell mediated immunity
adaptive immune response based on somatic recombination of immune receptors built from immunoglobulin superfamily domains
production of molecular mediator of immune response
adaptive immune response
lymphocyte mediated immunity
immune effector process
immunoglobulin mediated immune response
immunoglobulin production
leukocyte mediated immunity
B cell receptor signaling pathway
complement activation, classical pathway
complement activation
antimicrobial humoral response
antibacterial humoral response
antigen receptor−mediated signaling pathway
positive regulation of type I hypersensitivity
regulation of type I hypersensitivity
positive regulation of type II hypersensitivity
positive regulation of type IIa hypersensitivity
type I hypersensitivity
positive regulation of type I hypersensitivity
type II hypersensitivity
regulation of type II hypersensitivity
type IIa hypersensitivity
regulation of hypersensitivity
regulation of type IIa hypersensitivity
positive regulation of acute inflammatory response to antigenic stimulus
hypersensitivity
protein folding
protein maturation
protein folding
response to endoplasmic reticulum stress
ERAD pathway
response to endoplasmic reticulum stress
chaperone−mediated protein folding
protein folding in endoplasmic reticulum
response to topologically incorrect protein
to topologically incorrect protein
regulation of response to endoplasmic reticulum stress
protein exit from endoplasmic reticulum
protein localization to endoplasmic reticulum
vesicle−mediated transport between endosomal compartments
protein exit from endoplasmic reticulum
protein targeting to ER
reticulum to cytosol transport

ME25

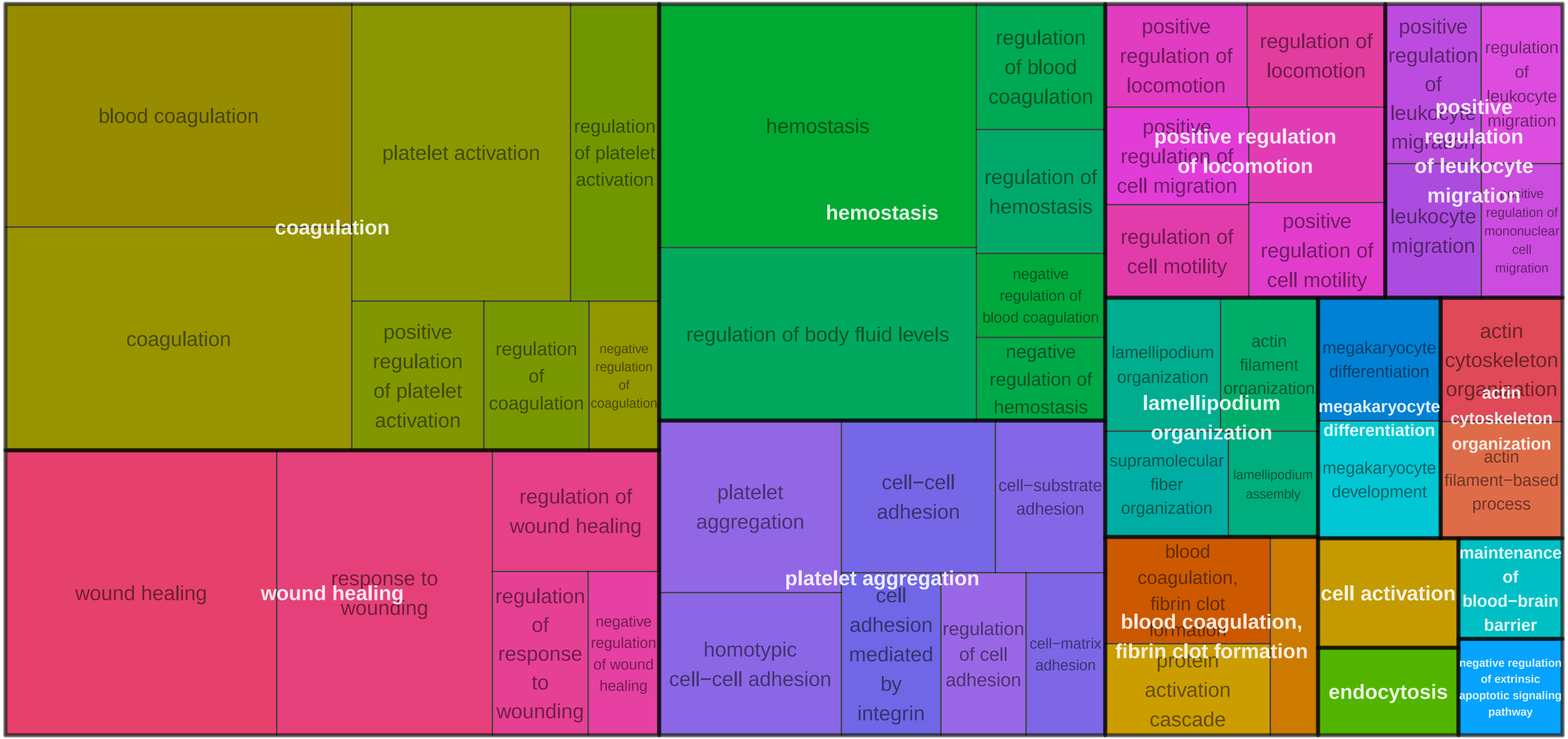
blood coagulation
platelet activation
regulation of platelet activation
coagulation
coagulation
positive regulation of platelet activation
regulation of coagulation
negative regulation of coagulation
hemostasis
hemostasis
regulation of blood coagulation
regulation of hemostasis
regulation of body fluid levels
negative regulation of blood coagulation
negative regulation of hemostasis
positive regulation of locomotion
regulation of locomotion
positive regulation of cell migration
positive regulation of locomotion
regulation of cell motility
positive regulation of cell motility
positive regulation of leukocyte migration
regulation of leukocyte migration
positive regulation of leukocyte migration
leukocyte migration
positive regulation of mononuclear cell migration
wound healing
wound healing
response to wounding
regulation of wound healing
regulation of response to wounding
negative regulation of wound healing
platelet aggregation
cell−cell adhesion
cell−substrate adhesion
platelet aggregation
homotypic cell−cell adhesion
cell adhesion mediated by integrin
regulation of cell adhesion
cell−matrix adhesion
lamellipodium organization
actin filament organization
lamellipodium organization
supramolecular fiber organization
lamellipodium assembly
megakaryocyte differentiation
megakaryocyte differentiation
megakaryocyte development
actin cytoskeleton organization
actin cytoskeleton organization
actin filament−based process
blood coagulation, fibrin clot formation
blood coagulation, fibrin clot formation
protein activation cascade
cell activation
maintenance of blood−brain barrier
endocytosis
negative regulation of extrinsic apoptotic signaling pathway

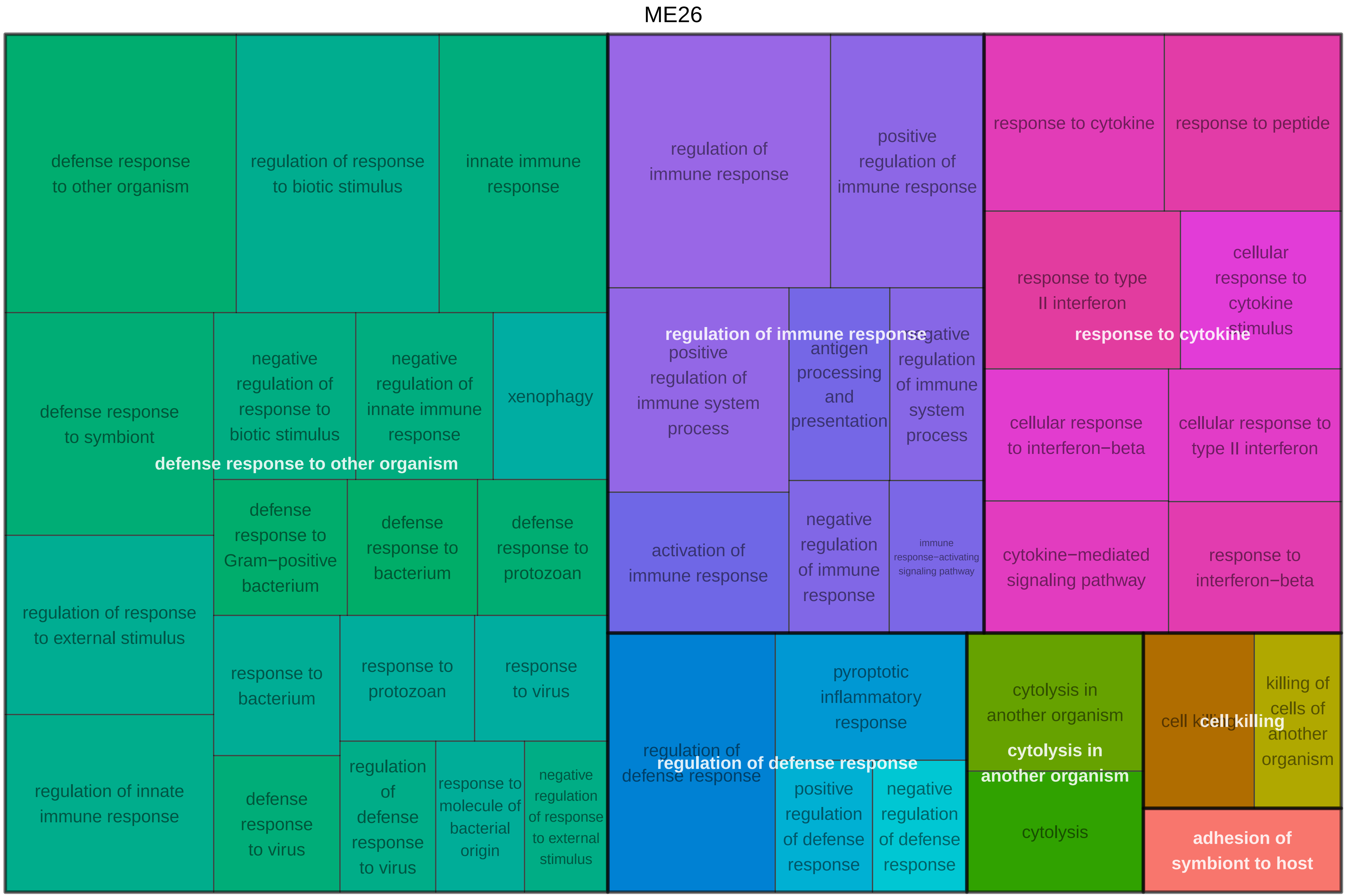
ME26
defense response to other organism
regulation of response to biotic stimulus
innate immune response
defense response to symbiont
negative regulation of response to biotic stimulus
negative regulation of innate immune response
xenophagy
defense response to other organism
defense response to Gram−positive bacterium
defense response to bacterium
defense response to protozoan
regulation of response to external stimulus
response to bacterium
response to protozoan
response to virus
regulation of innate immune response
defense response to virus
regulation of defense response to virus
response to molecule of bacterial origin
negative regulation of response to external stimulus
regulation of immune response
positive regulation of immune response
regulation of immune response
positive regulation of immune system process
antigen processing and presentation
negative regulation of immune system process
activation of immune response
negative regulation of immune response
immune response−activating signaling pathway
response to cytokine
response to peptide
response to type II interferon
cellular response to cytokine stimulus
response to cytokine
cellular response to interferon−beta
cellular response to type II interferon
cytokine−mediated signaling pathway
response to interferon−beta
regulation of defense response
pyroptotic inflammatory response
regulation of defense response
positive regulation of defense response
negative regulation of defense response
cytolysis in another organism
cytolysis in another organism
cytolysis
cell killing
cell killing
killing of cells of another organism
adhesion of symbiont to host

ME28

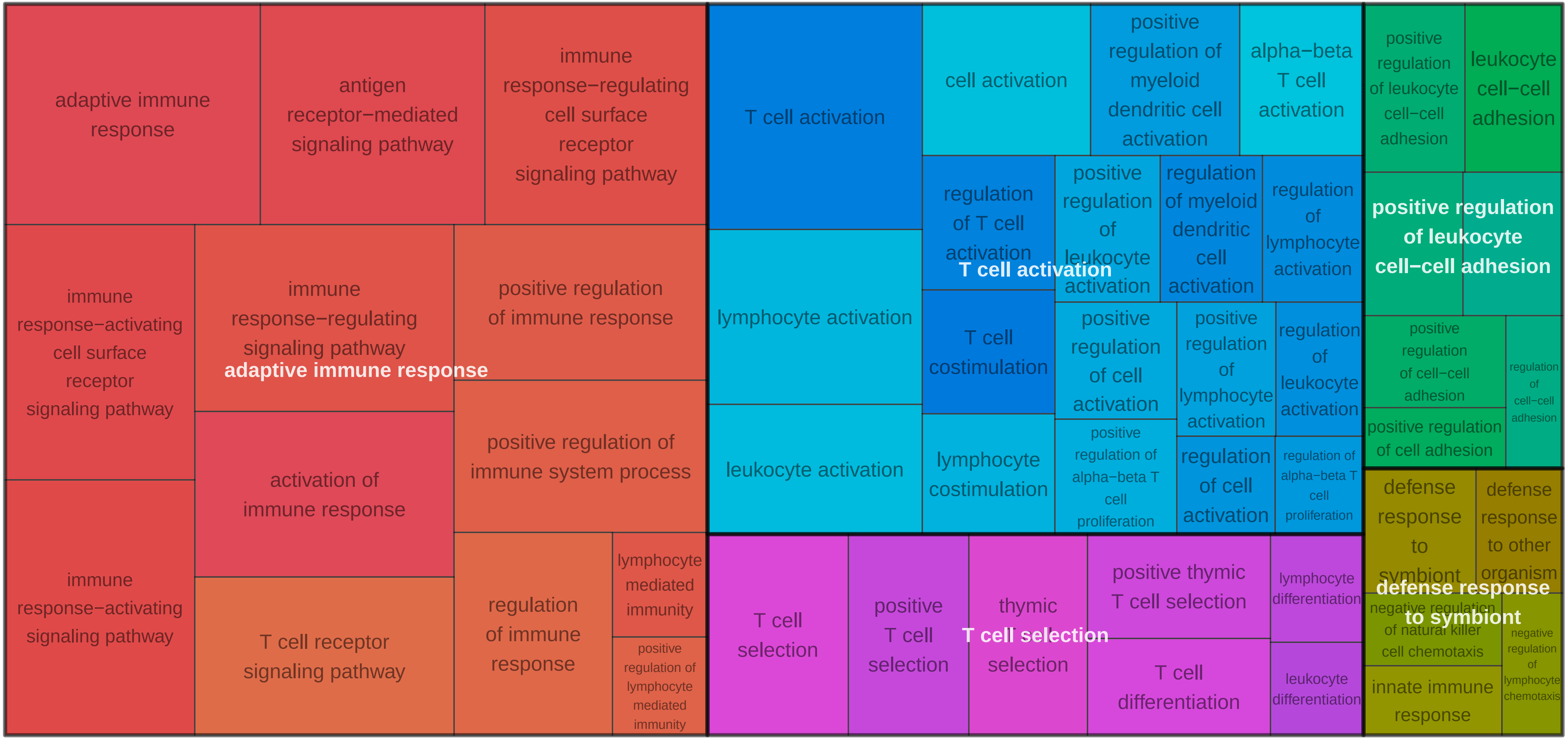
adaptive immune response
antigen receptor−mediated signaling pathway
immune response−regulating cell surface receptor signaling pathway
immune response−activating cell surface receptor signaling pathway
immune response−regulating signaling pathway
adaptive immune response
positive regulation of immune response
activation of immune response
positive regulation of immune system process
immune response−activating signaling pathway
T cell receptor signaling pathway
regulation of immune response
lymphocyte mediated immunity
positive regulation of lymphocyte mediated immunity
T cell activation
cell activation
positive regulation of myeloid dendritic cell activation
alpha−beta T cell activation
regulation of T cell activation
T cell activation
positive regulation of leukocyte activation
regulation of myeloid dendritic cell activation
regulation of lymphocyte activation
lymphocyte activation
T cell costimulation
positive regulation of cell activation
positive regulation of lymphocyte activation
regulation of leukocyte activation
leukocyte activation
lymphocyte costimulation
positive regulation of alpha−beta T cell proliferation
regulation of cell activation
regulation of alpha−beta T cell proliferation
positive regulation of leukocyte cell−cell adhesion
leukocyte cell−cell adhesion
positive regulation of leukocyte cell−cell adhesion
positive regulation of cell−cell adhesion
regulation of cell−cell adhesion
positive regulation of cell adhesion
defense response to symbiont
defense response to other organism
defense response to symbiont
negative regulation of natural killer cell chemotaxis
negative regulation of lymphocyte chemotaxis
innate immune response
T cell selection
positive T cell selection
thymic T cell selection
T cell selection
positive thymic T cell selection
lymphocyte differentiation
T cell differentiation
leukocyte differentiation

ME3

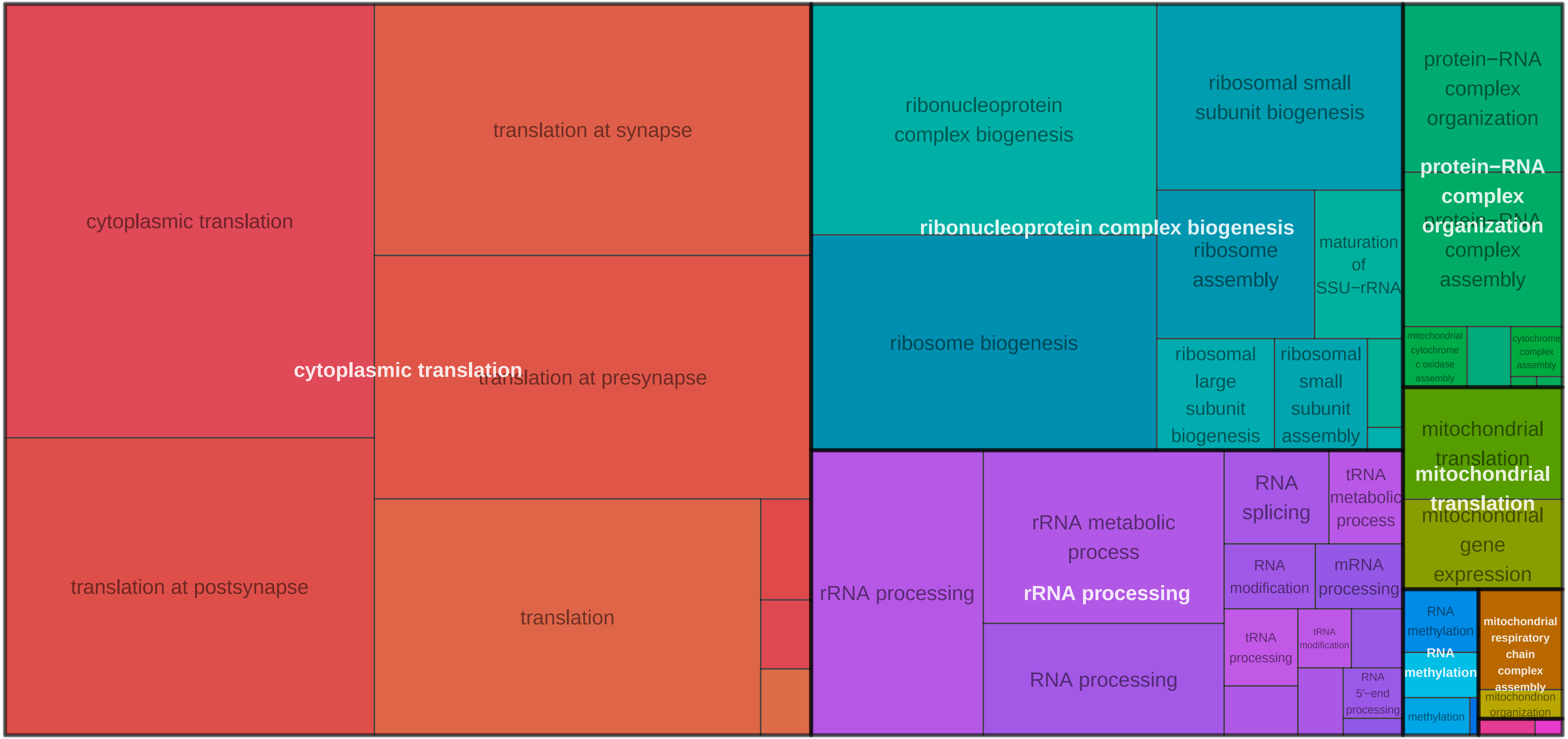

cytoplasmic translation
translation at synapse
cytoplasmic translation
translation at presynapse
translation at postsynapse
translation
ribonucleoprotein complex biogenesis
ribosomal small subunit biogenesis
ribonucleoprotein complex biogenesis
ribosome assembly
maturation of SSU−rRNA
ribosome biogenesis
ribosomal large subunit biogenesis
ribosomal small subunit assembly
protein−RNA complex organization
protein−RNA complex organization
protein−RNA complex assembly
mitochondrial cytochrome c oxidase assembly
cytochrome complex assembly
mitochondrial translation
mitochondrial translation
mitochondrial gene expression
rRNA processing
rRNA metabolic process
rRNA processing
RNA splicing
tRNA metabolic process
RNA modification
mRNA processing
RNA processing
tRNA processing
tRNA modification
RNA 5'−end processing
RNA methylation
RNA methylation
methylation
mitochondrial respiratory chain complex assembly
mitochondrion organization

ME32

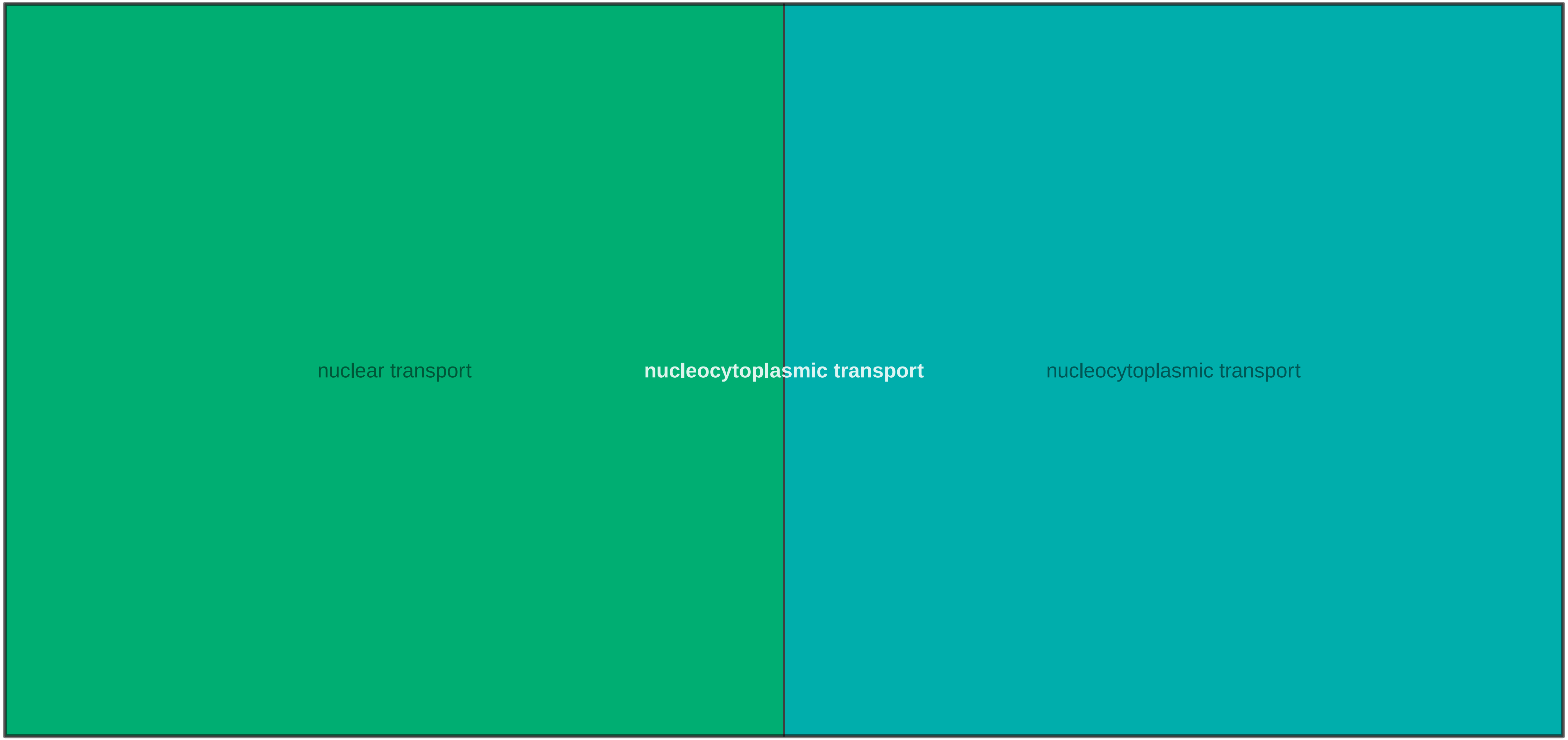
nuclear transport
nucleocytoplasmic transport
nucleocytoplasmic transport

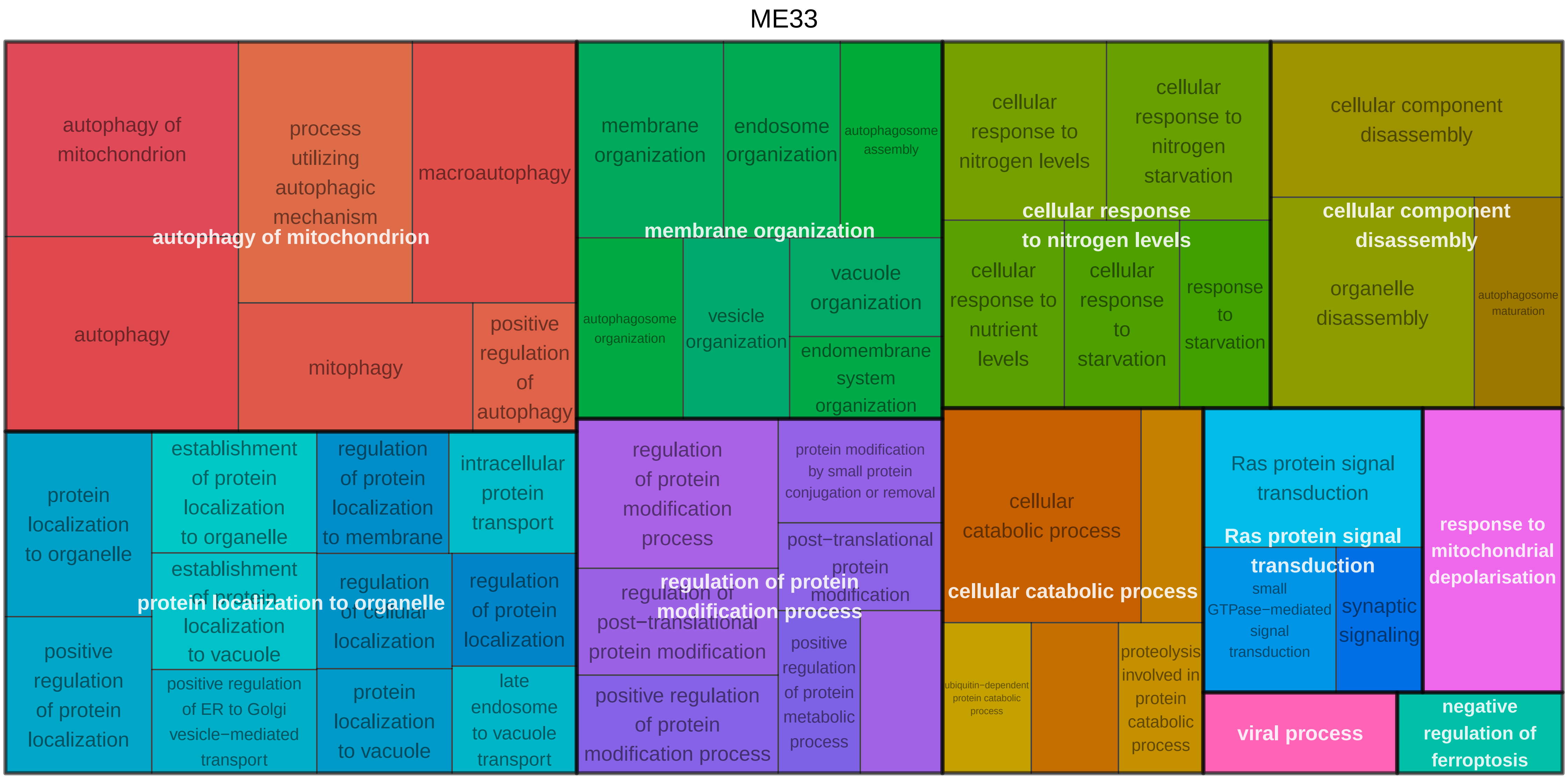
ME33
autophagy of mitochondrion
process utilizing autophagic mechanism
macroautophagy
autophagy of mitochondrion
autophagy
mitophagy
positive regulation of autophagy
membrane organization
endosome organization
autophagosome assembly
membrane organization
autophagosome organization
vesicle organization
vacuole organization
endomembrane system organization
cellular response to nitrogen levels
cellular response to nitrogen starvation
cellular response to nitrogen levels
cellular response to nutrient levels
cellular response to starvation
response to starvation
cellular component disassembly
cellular component disassembly
organelle disassembly
autophagosome maturation
protein localization to organelle
establishment of protein localization to organelle
regulation of protein localization to membrane
intracellular protein transport
establishment of protein localization to vacuole
protein localization to organelle
regulation of cellular localization
regulation of protein localization
positive regulation of protein localization
positive regulation of ER to Golgi vesicle−mediated transport
protein localization to vacuole
late endosome to vacuole transport
regulation of protein modification process
protein modification by small protein conjugation or removal
post−translational protein modification
regulation of post−translational protein modification
regulation of protein modification process
positive regulation of protein modification process
positive regulation of protein metabolic process
cellular catabolic process
cellular catabolic process
ubiquitin−dependent protein catabolic process
proteolysis involved in protein catabolic process
Ras protein signal transduction
Ras protein signal transduction
small GTPase−mediated signal transduction
synaptic signaling
response to mitochondrial depolarisation
viral process
negative regulation of ferroptosis

ME34

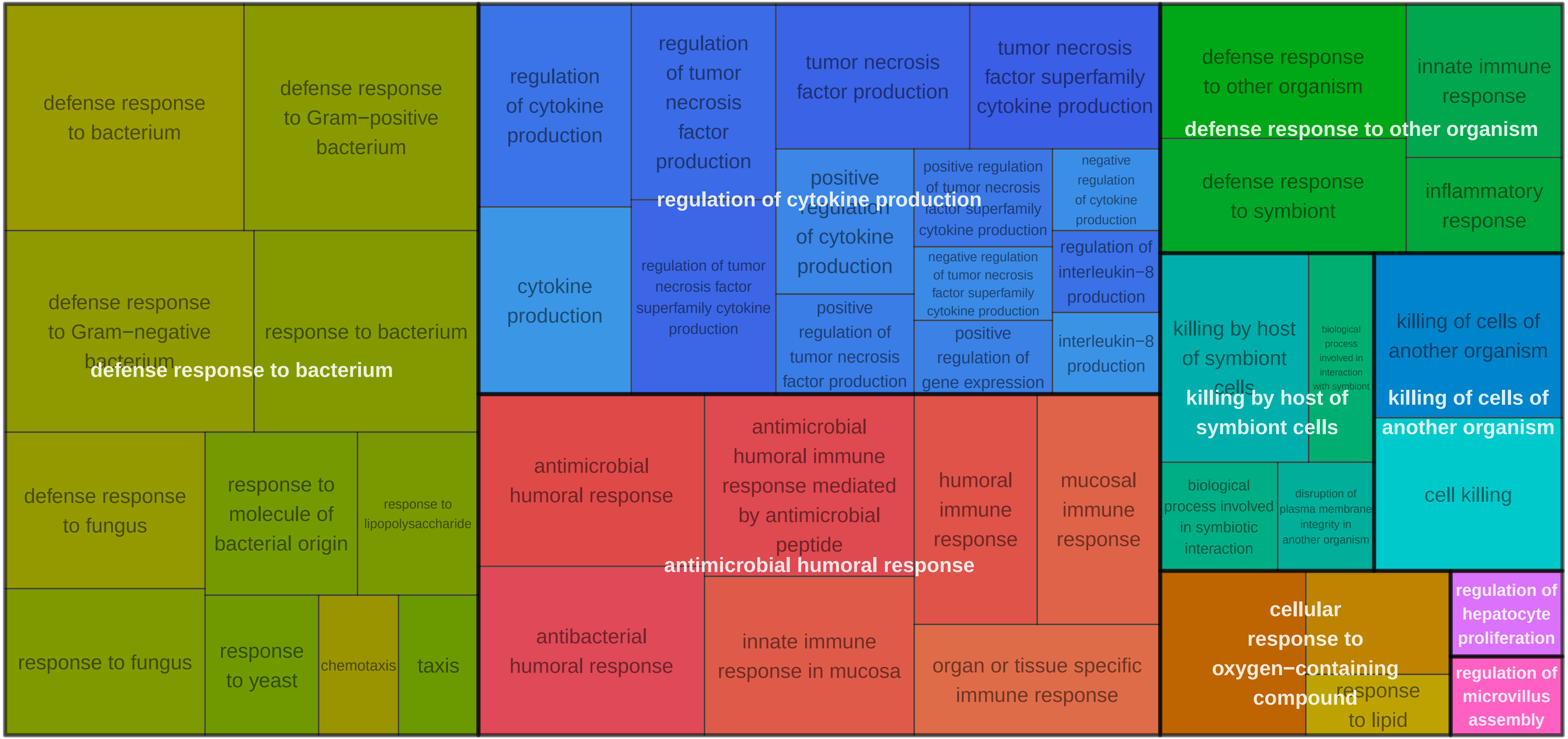

defense response to bacterium
defense response to Gram−positive bacterium
defense response to Gram−negative bacterium
response to bacterium
defense response to bacterium
defense response to fungus
response to molecule of bacterial origin
response to lipopolysaccharide
response to fungus
response to yeast
chemotaxis
taxis
regulation of cytokine production
regulation of tumor necrosis factor production
tumor necrosis factor production
tumor necrosis factor superfamily cytokine production
cytokine production
regulation of tumor necrosis factor superfamily cytokine production
positive regulation of cytokine production
positive regulation of tumor necrosis factor superfamily cytokine production
negative regulation of cytokine production
regulation of cytokine production
negative regulation of tumor necrosis factor superfamily cytokine production
regulation of interleukin−8 production
positive regulation of tumor necrosis factor production
positive regulation of gene expression
interleukin−8 production
defense response to other organism
innate immune response
defense response to other organism
defense response to symbiont
inflammatory response
killing by host of symbiont cells
biological process involved in interaction with symbiont
killing by host of symbiont cells
killing of cells of another organism
killing of cells of another organism
biological process involved in symbiotic interaction
disruption of plasma membrane integrity in another organism
cell killing
antimicrobial humoral response
antimicrobial humoral immune response mediated by antimicrobial peptide
humoral immune response
mucosal immune response
antimicrobial humoral response
antibacterial humoral response
innate immune response in mucosa
organ or tissue specific immune response
cellular response to oxygen−containing compound
response to lipid
regulation of hepatocyte proliferation
regulation of microvillus assembly

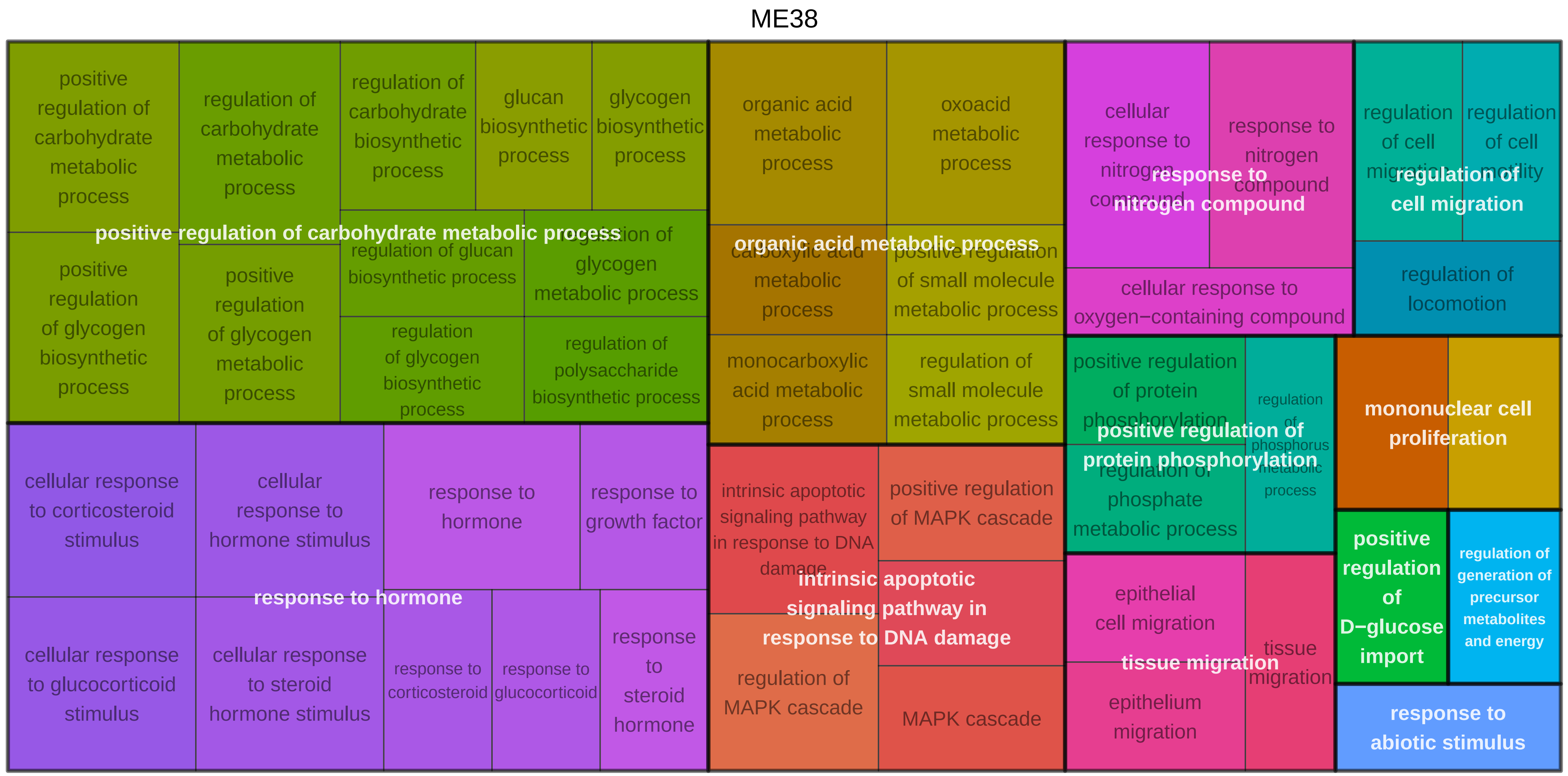
ME38
positive regulation of carbohydrate metabolic process
positive regulation of carbohydrate metabolic process
regulation of carbohydrate metabolic process
regulation of carbohydrate biosynthetic process
glucan biosynthetic process
glycogen biosynthetic process
positive regulation of glycogen biosynthetic process
positive regulation of glycogen metabolic process
regulation of glucan biosynthetic process
regulation of glycogen metabolic process
regulation of glycogen biosynthetic process
regulation of polysaccharide biosynthetic process
organic acid metabolic process
organic acid metabolic process
oxoacid metabolic process
carboxylic acid metabolic process
positive regulation of small molecule metabolic process
monocarboxylic acid metabolic process
regulation of small molecule metabolic process
response to nitrogen compound
cellular response to nitrogen compound
response to nitrogen compound
cellular response to oxygen−containing compound
regulation of cell migration
regulation of cell migration
regulation of cell motility
regulation of locomotion
positive regulation of protein phosphorylation
positive regulation of protein phosphorylation
regulation of phosphorus metabolic process
regulation of phosphate metabolic process
mononuclear cell proliferation
response to hormone
cellular response to corticosteroid stimulus
cellular response to hormone stimulus
response to hormone
response to growth factor
cellular response to glucocorticoid stimulus
cellular response to steroid hormone stimulus
response to corticosteroid
response to glucocorticoid
response to steroid hormone
intrinsic apoptotic signaling pathway in response to DNA damage
intrinsic apoptotic signaling pathway in response to DNA damage
positive regulation of MAPK cascade
regulation of MAPK cascade
MAPK cascade
tissue migration
epithelial cell migration
tissue migration
epithelium migration
positive regulation of D−glucose import
regulation of generation of precursor metabolites and energy
response to abiotic stimulus

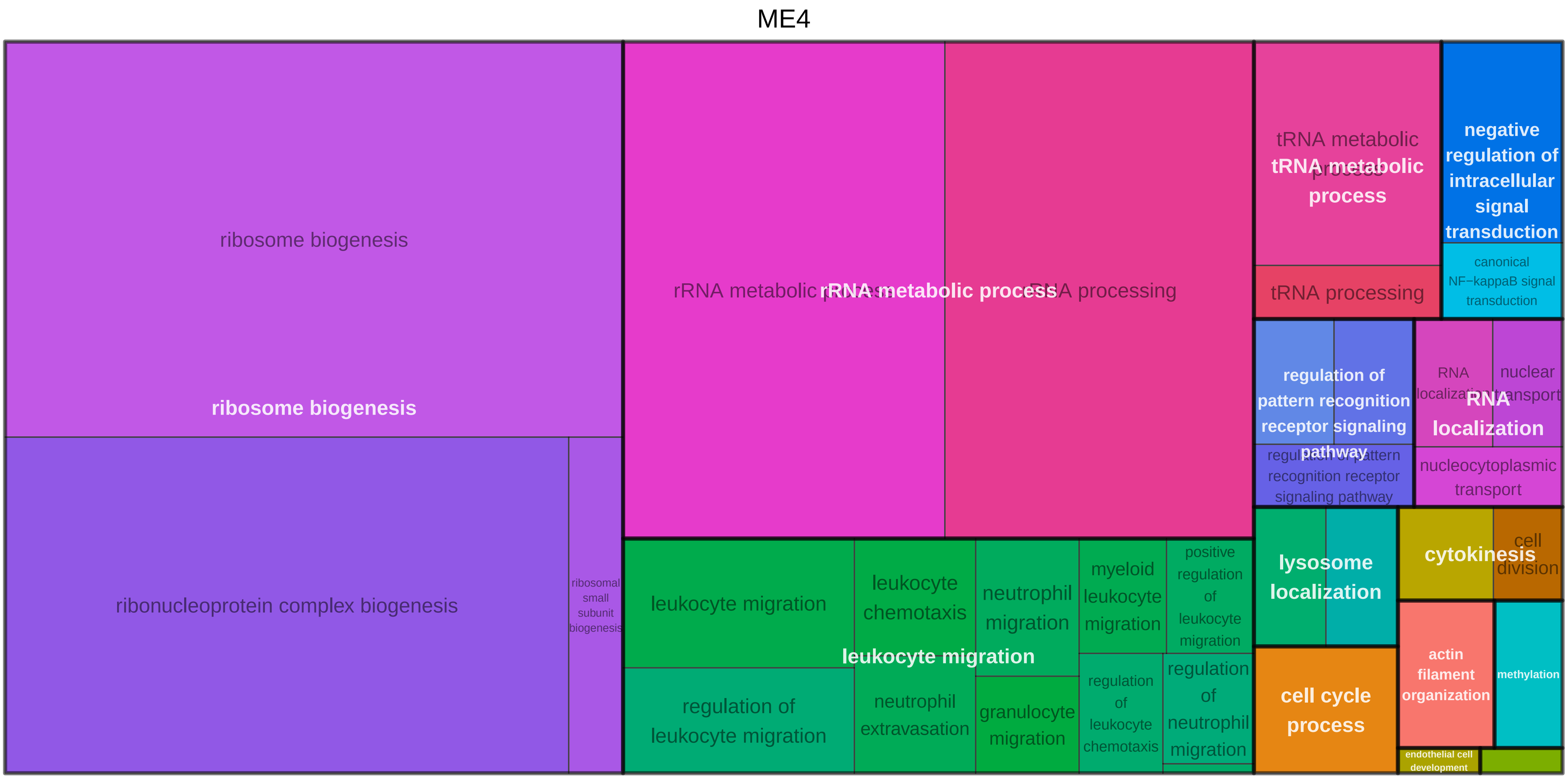
ME4
ribosome biogenesis
ribosome biogenesis
ribonucleoprotein complex biogenesis
ribosomal small subunit biogenesis
rRNA metabolic process
rRNA metabolic process
rRNA processing
tRNA metabolic process
tRNA metabolic process
tRNA processing
negative regulation of intracellular signal transduction
canonical NF−kappaB signal transduction
regulation of pattern recognition receptor signaling pathway
regulation of pattern recognition receptor signaling pathway
RNA localization
nuclear transport
RNA localization
nucleocytoplasmic transport
leukocyte migration
leukocyte chemotaxis
neutrophil migration
myeloid leukocyte migration
positive regulation of leukocyte migration
leukocyte migration
regulation of leukocyte migration
neutrophil extravasation
granulocyte migration
regulation of leukocyte chemotaxis
regulation of neutrophil migration
lysosome localization
cytokinesis
cell division
cell cycle process
actin filament organization
methylation
endothelial cell development

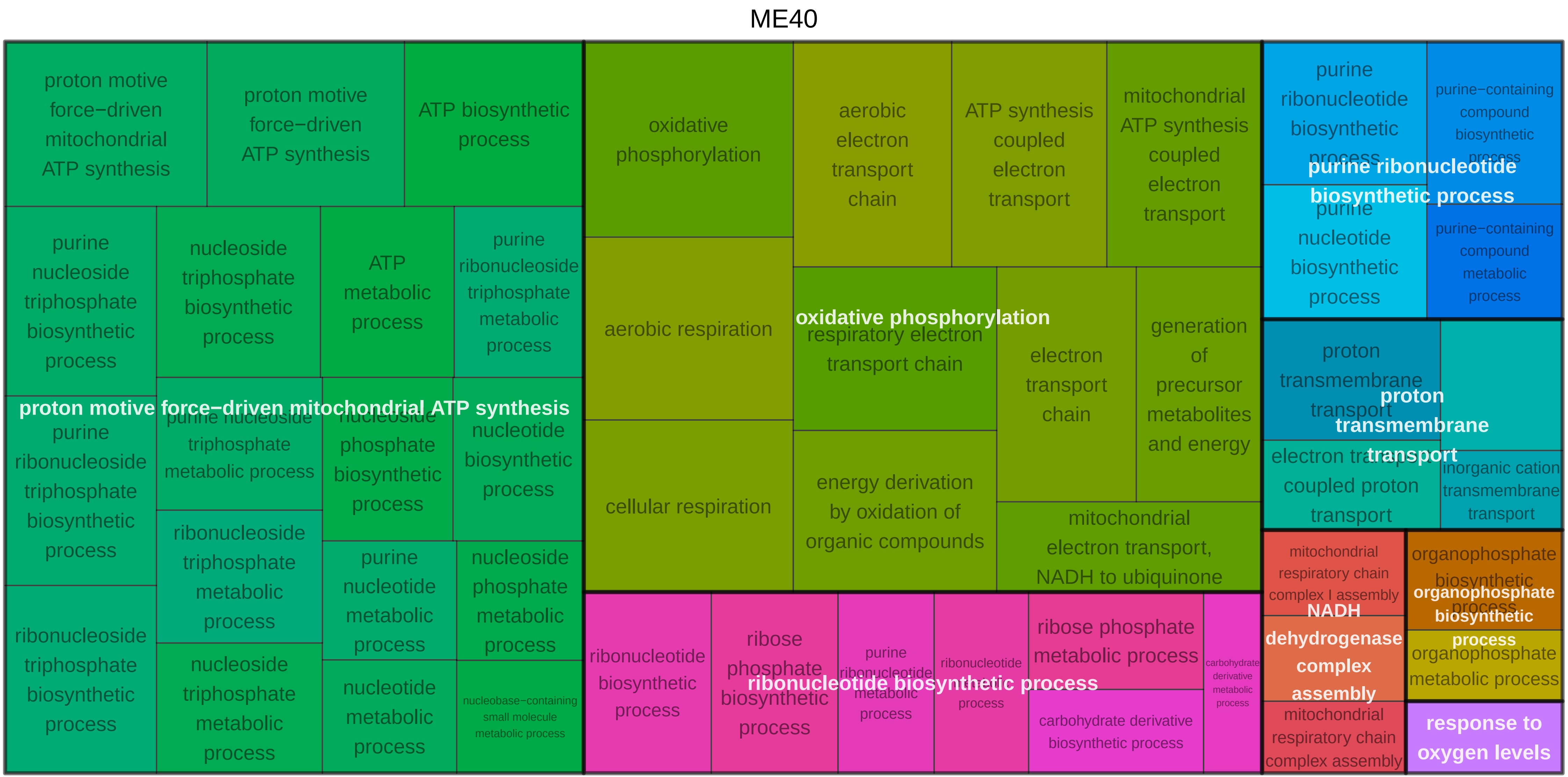
ME40
proton motive force−driven mitochondrial ATP synthesis
proton motive force−driven ATP synthesis
ATP biosynthetic process
purine nucleoside triphosphate biosynthetic process
nucleoside triphosphate biosynthetic process
ATP metabolic process
purine ribonucleoside triphosphate metabolic process
purine ribonucleoside triphosphate biosynthetic process
purine nucleoside triphosphate metabolic process
nucleoside phosphate biosynthetic process
nucleotide biosynthetic process
ribonucleoside triphosphate metabolic process
purine nucleotide metabolic process
nucleoside phosphate metabolic process
ribonucleoside triphosphate biosynthetic process
nucleoside triphosphate metabolic process
nucleotide metabolic process
nucleobase−containing small molecule metabolic process
proton motive force−driven mitochondrial ATP synthesis
oxidative phosphorylation
aerobic electron transport chain
ATP synthesis coupled electron transport
mitochondrial ATP synthesis coupled electron transport
aerobic respiration
respiratory electron transport chain
electron transport chain
generation of precursor metabolites and energy
oxidative phosphorylation
cellular respiration
energy derivation by oxidation of organic compounds
mitochondrial electron transport, NADH to ubiquinone
purine ribonucleotide biosynthetic process
purine−containing compound biosynthetic process
purine ribonucleotide biosynthetic process
purine nucleotide biosynthetic process
purine−containing compound metabolic process
proton transmembrane transport
proton transmembrane transport
electron transport coupled proton transport
inorganic cation transmembrane transport
ribonucleotide biosynthetic process
ribose phosphate biosynthetic process
purine ribonucleotide metabolic process
ribonucleotide metabolic process
ribose phosphate metabolic process
carbohydrate derivative metabolic process
carbohydrate derivative biosynthetic process
ribonucleotide biosynthetic process
mitochondrial respiratory chain complex I assembly
NADH dehydrogenase complex assembly
mitochondrial respiratory chain complex assembly
organophosphate biosynthetic process
organophosphate biosynthetic process
organophosphate metabolic process
response to oxygen levels

ME7

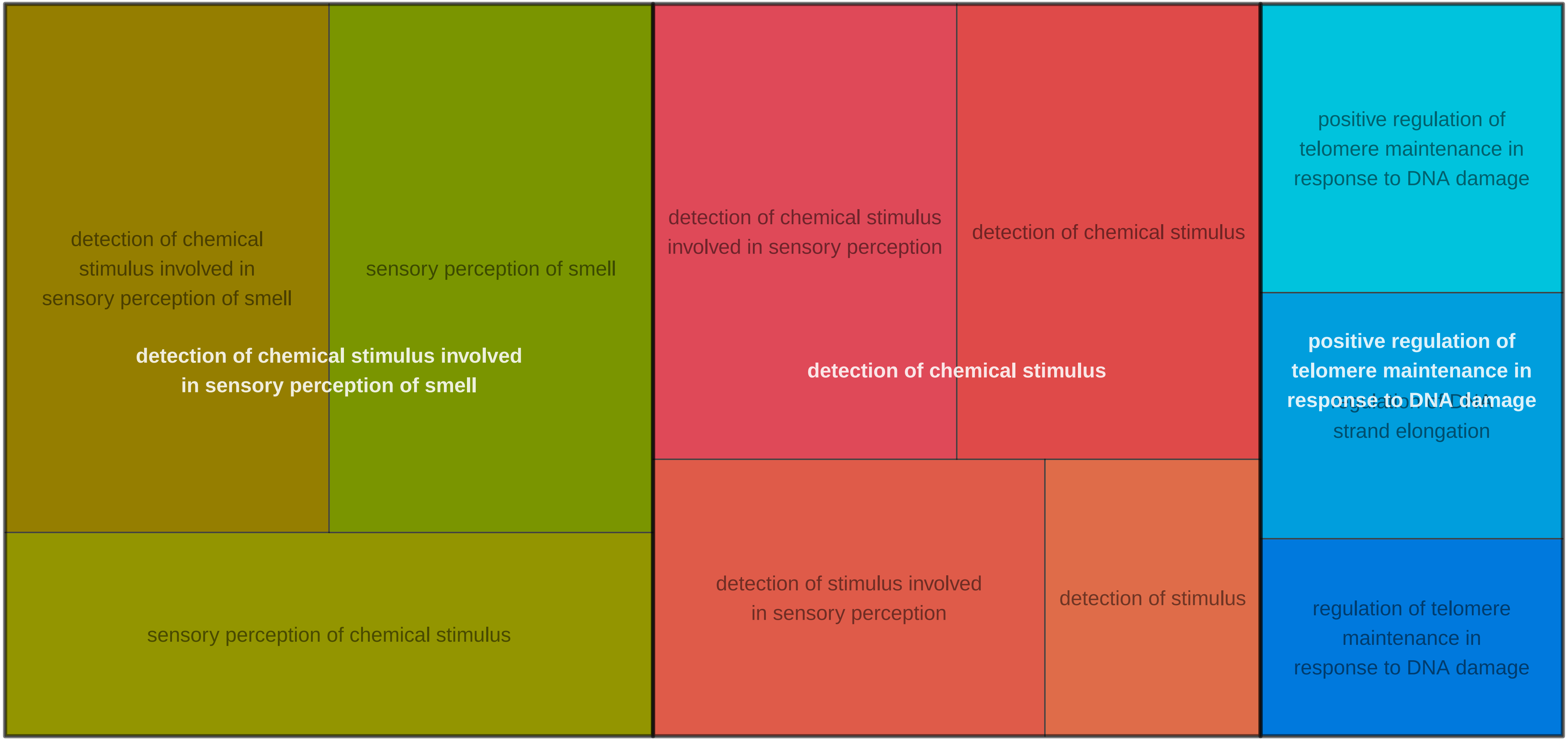
detection of chemical stimulus involved in sensory perception of smell
sensory perception of smell
detection of chemical stimulus involved in sensory perception of smell
sensory perception of chemical stimulus
detection of chemical stimulus involved in sensory perception
detection of chemical stimulus
detection of chemical stimulus
detection of stimulus involved in sensory perception
detection of stimulus
positive regulation of telomere maintenance in response to DNA damage
positive regulation of telomere maintenance in response to DNA damage
strand elongation
regulation of telomere maintenance in response to DNA damage

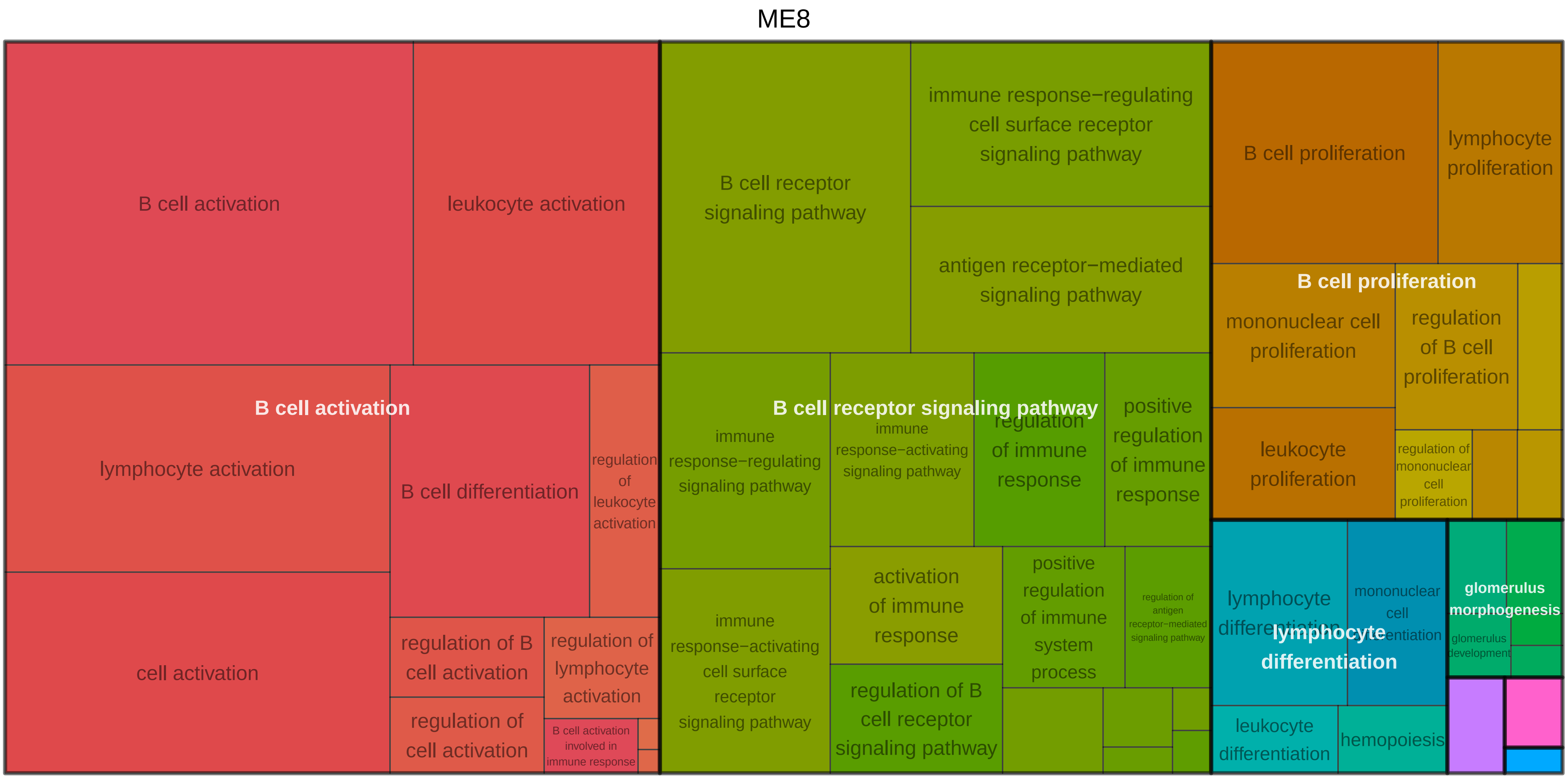

ME8
B cell activation
leukocyte activation
B cell activation
lymphocyte activation
B cell differentiation
regulation of leukocyte activation
cell activation
regulation of B cell activation
regulation of lymphocyte activation
regulation of cell activation
B cell activation involved in immune response
B cell receptor signaling pathway
immune response−regulating cell surface receptor signaling pathway
antigen receptor−mediated signaling pathway
B cell receptor signaling pathway
immune response−regulating signaling pathway
immune response−activating signaling pathway
regulation of immune response
positive regulation of immune response
immune response−activating cell surface receptor signaling pathway
activation of immune response
positive regulation of immune system process
regulation of antigen receptor−mediated signaling pathway
regulation of B cell receptor signaling pathway
B cell proliferation
lymphocyte proliferation
B cell proliferation
mononuclear cell proliferation
regulation of B cell proliferation
leukocyte proliferation
regulation of mononuclear cell proliferation
lymphocyte differentiation
mononuclear cell differentiation
lymphocyte differentiation
leukocyte differentiation
hemopoiesis
glomerulus morphogenesis
glomerulus development

ME9

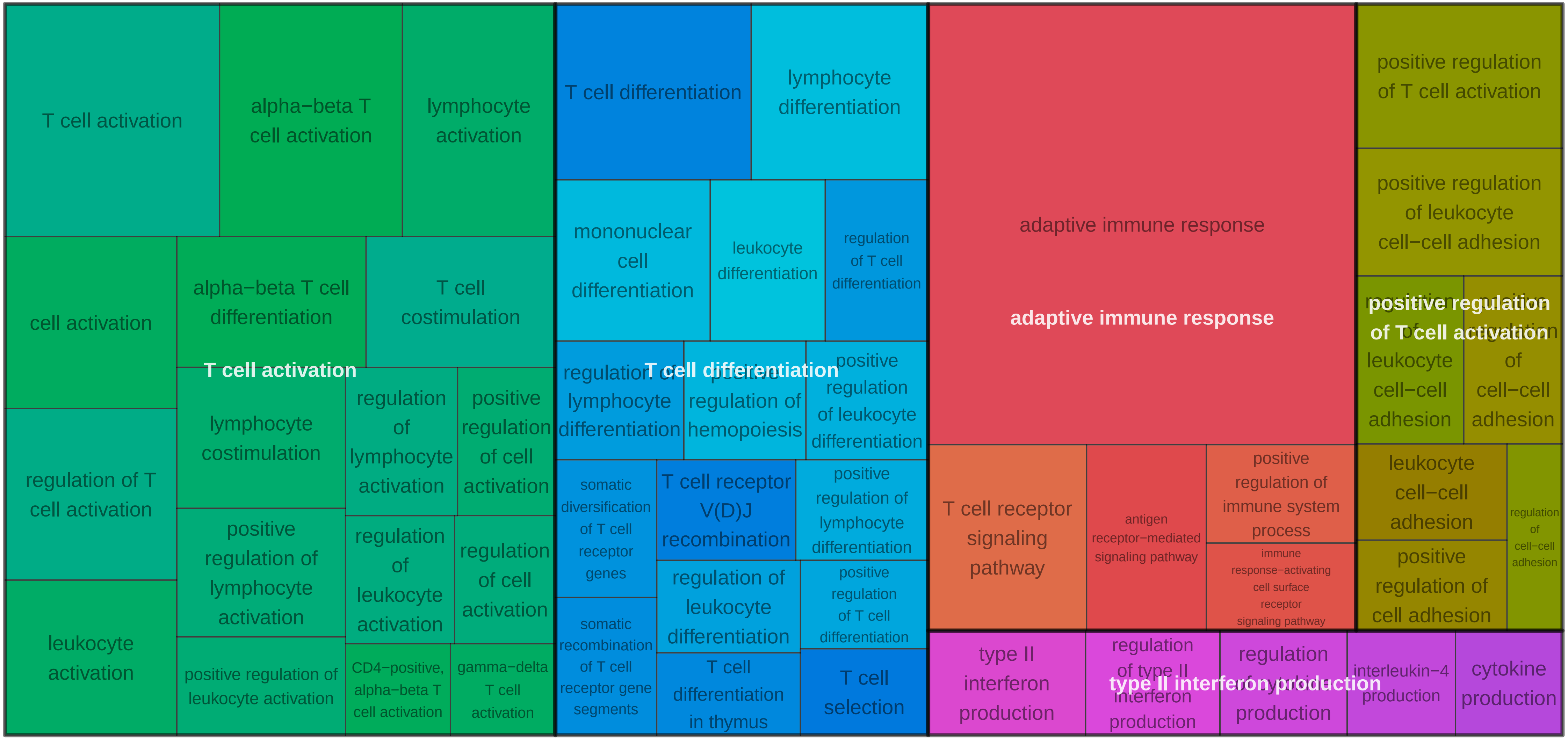
T cell activation
alpha−beta T cell activation
lymphocyte activation
cell activation
alpha−beta T cell differentiation
T cell costimulation
T cell activation
lymphocyte costimulation
regulation of lymphocyte activation
positive regulation of cell activation
regulation of T cell activation
positive regulation of lymphocyte activation
regulation of leukocyte activation
regulation of cell activation
leukocyte activation
positive regulation of leukocyte activation
CD4−positive, alpha−beta T cell activation
gamma−delta T cell activation
T cell differentiation
lymphocyte differentiation
mononuclear cell differentiation
leukocyte differentiation
regulation of T cell differentiation
T cell differentiation
lymphocyte differentiation
regulation of hemopoiesis
positive regulation of leukocyte differentiation
somatic diversification of T cell receptor genes
T cell receptor V(D)J recombination
positive regulation of lymphocyte differentiation
regulation of leukocyte differentiation
positive regulation of T cell differentiation
somatic recombination of T cell receptor gene segments
T cell differentiation in thymus
T cell selection
adaptive immune response
adaptive immune response
T cell receptor signaling pathway
antigen receptor−mediated signaling pathway
positive regulation of immune system process
immune response−activating cell surface receptor signaling pathway
type II interferon production
regulation of type II interferon production
type II interferon production
regulation of
production
interleukin−4 production
cytokine production
positive regulation of T cell activation
positive regulation of leukocyte cell−cell adhesion
positive regulation of T cell activation
leukocyte cell−cell adhesion
of cell−cell adhesion
leukocyte cell−cell adhesion
regulation of cell−cell adhesion
positive regulation of cell adhesion

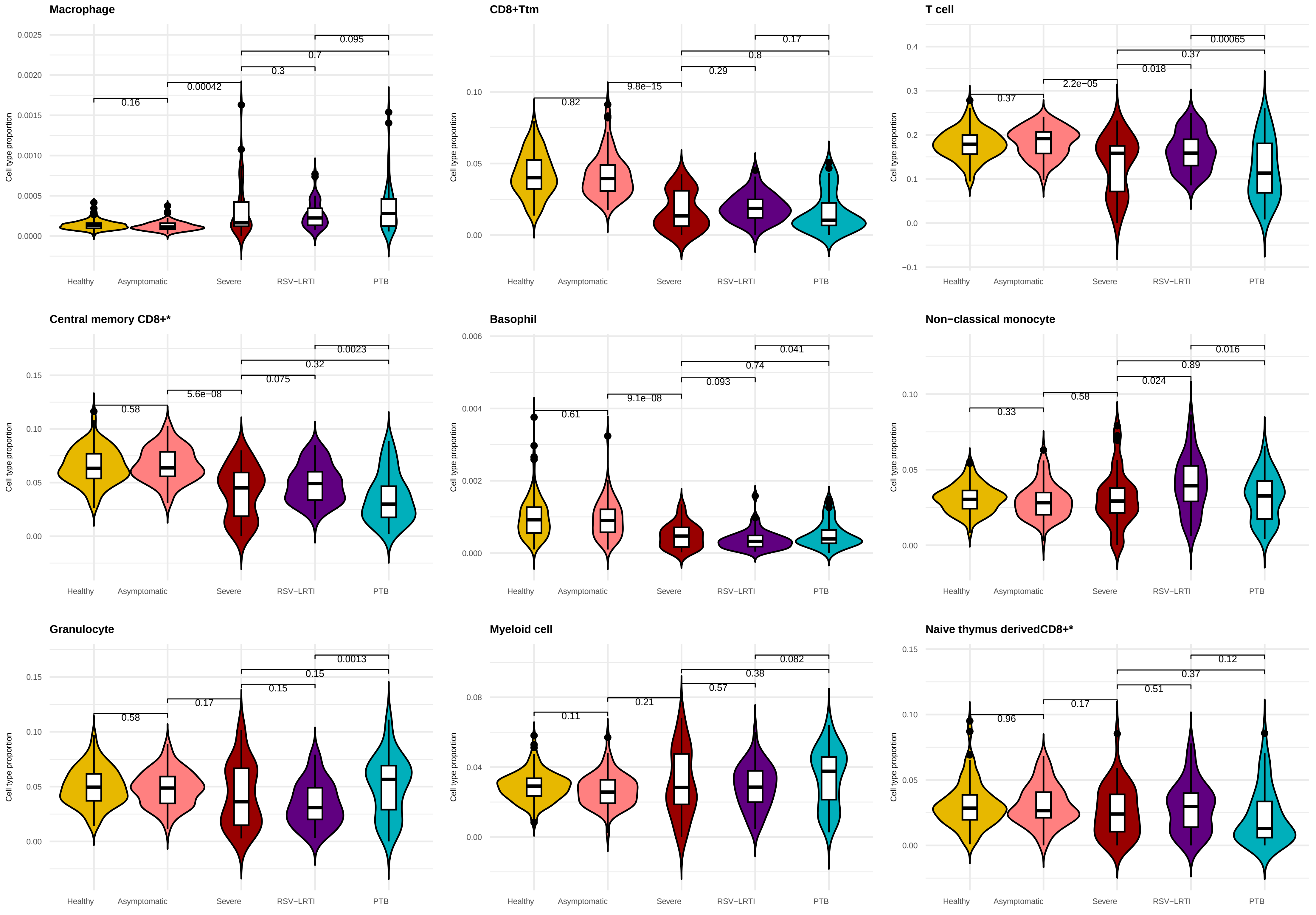

Macrophage
Cell type proportion
0.0025
0.0020
0.0015
0.0010
0.0005
0.0000
0.095
0.7
0.3
0.00042
0.16
Healthy
Asymptomatic
Severe
RSV−LRTI
PTB
CD8+Ttm
Cell type proportion
0.10
0.05
0.00
0.17
0.8
0.29
9.8e−15
0.82
Healthy
Asymptomatic
Severe
RSV−LRTI
PTB
T cell
Cell type proportion
0.4
0.3
0.2
0.1
0.0
−0.1
0.00065
0.37
0.018
2.2e−05
0.37
Healthy
Asymptomatic
Severe
RSV−LRTI
PTB
Central memory CD8+*
Cell type proportion
0.15
0.10
0.05
0.00
0.0023
0.32
0.075
5.6e−08
0.58
Healthy
Asymptomatic
Severe
RSV−LRTI
PTB
Basophil
Cell type proportion
0.006
0.004
0.002
0.000
0.041
0.74
0.093
9.1e−08
0.61
Healthy
Asymptomatic
Severe
RSV−LRTI
PTB
Non−classical monocyte
Cell type proportion
0.10
0.05
0.00
0.016
0.89
0.024
0.58
0.33
Healthy
Asymptomatic
Severe
RSV−LRTI
PTB
Granulocyte
Cell type proportion
0.15
0.10
0.05
0.00
0.0013
0.15
0.15
0.17
0.58
Healthy
Asymptomatic
Severe
RSV−LRTI
PTB
Myeloid cell
Cell type proportion
0.08
0.04
0.00
0.082
0.38
0.57
0.21
0.11
Healthy
Asymptomatic
Severe
RSV−LRTI
PTB
Naive thymus derivedCD8+*
Cell type proportion
0.15
0.10
0.05
0.00
0.12
0.37
0.51
0.17
0.96
Healthy
Asymptomatic
Severe
RSV−LRTI
PTB

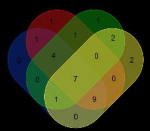
1
1
1
1
2
0
4
0
2
0
7
0
1
9
0

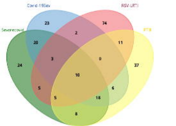
Covid-19Sev
RSV-LRTI
Severecovid
PTB
23
74
20
2
11
24
3
0
37
10
5
5
18
6
8
